# Evaluation of Amino-Functional Polyester Dendrimers Based on Bis-MPA as Nonviral Vectors for siRNA Delivery

**DOI:** 10.3390/molecules23082028

**Published:** 2018-08-14

**Authors:** Patrik Stenström, Dario Manzanares, Yuning Zhang, Valentin Ceña, Michael Malkoch

**Affiliations:** 1 Fiber and Polymer Technology, KTH Royal Institute of Technology, 10044 Stockholm, Sweden; pstens@kth.se (P.S.); yunzha@kth.se (Y.Z.); 2 Unidad Asociada Neurodeath, Universidad de Castilla-La Mancha, 02006 Albacete, Spain; dariomanzafarmacia@gmail.com (D.M.); valentin.cena@gmail.com (V.C.); 3 CIBERNED, Instituto de Salud Carlos III, 28029 Madrid, Spain

**Keywords:** dendrimer, siRNA, RNAi, bis-MPA, monodisperse, polycation

## Abstract

Herein, we present the first evaluation of cationic dendrimers based on 2,2-bis(methylol)propionic acid (bis-MPA) as nonviral vectors for transfection of short interfering RNA (siRNA) in cell cultures. The study encompassed dendrimers of generation one to four (G1–G4), modified to bear 6–48 amino end-groups, where the G2–G4 proved to be capable of siRNA complexation and protection against RNase-mediated degradation. The dendrimers were nontoxic to astrocytes, glioma (C6), and glioblastoma (U87), while G3 and G4 exhibited concentration dependent toxicity towards primary neurons. The G2 showed no toxicity to primary neurons at any of the tested concentrations. Fluorescence microscopy experiments suggested that the dendrimers are highly efficient at endo-lysosomal escape since fluorescently labeled dendrimers were localized specifically in mitochondria, and diffuse cytosolic distribution of fluorescent siRNA complexed by dendrimers was observed. This is a desired feature for intracellular drug delivery, since the endocytic pathway otherwise transfers the drugs into lysosomes where they can be degraded without reaching their intended target. siRNA-transfection was successful in C6 and U87 cell lines using the G3 and G4 dendrimers followed by a decrease of approximately 20% of target protein p42-MAPK expression.

## 1. Introduction

In 1998 researchers found that short sequences of double-stranded RNA could selectively degrade target mRNA through RNA interference (RNAi) and thus prevent the expression of disease-causing proteins [1]. This discovery awarded the authors Andrew Z. Fire and Craig C. Mello the Nobel Prize in 2006. Since nearly any protein-coding gene in the body can theoretically be targeted, the technique is applicable to a much broader array of diseases than those treatable with conventional drugs. These short RNA sequences of 21–23 bases were later termed short interfering RNAs (siRNAs), and are presently being investigated in clinical trials as potential treatments for cancer [2,3] and transthyretin amyloidosis (ATTR) [4,5], a life-threatening disease with cardiac and peripheral nerve symptoms.

However, some major obstacles have to be overcome to attain a successful treatment using siRNA. Free siRNA is rapidly degraded by ribonucleases (RNases) [6], and can be recognized by the immune system to cause inflammatory response [7]. The greatest challenge is perhaps the cellular uptake due to its large size and negative charge. RNAi can only occur through the formation of the RNA-induced silencing complex (RISC) [8], which takes place in cytoplasmic bodies called processing bodies (P-bodies) that are located throughout the cytoplasm [9]. Thus, it is necessary for the siRNA and its carrier to efficiently escape the endocytic pathway. These obstacles and examples of strategies for overcoming them have been extensively reviewed [10,11,12].

Viral vectors are generally considered to be the most efficient carriers for nucleic acid delivery. However, these are still struggling to achieve clinical approval due to problems with immune responses and other safety concerns, as well as their complexity of preparation [13,14,15]. This has led the scientific community towards the development of synthetic alternatives to viral vectors such as liposomes [16], polymers [17], and other nanoparticles [18].

Transfection of neuronal cells can be especially challenging [19,20]. Neurons are generally sensitive to alterations in their environment, and in vivo the brain is separated from the blood stream by the blood–brain barrier (BBB). Successful transfection techniques include the use of liposomes [16], electroporation [21], and viral vectors [22,23]. However, these techniques suffer from drawbacks such as immunological concerns, low transfection rates, or complex methodology.

Dendrimers, being highly branched and compact scaffolds, have been proposed as promising candidates for transporting siRNA inside cells and protecting it from degradation while travelling through the body or staying in the extracellular medium [24]. Cationic dendrimers of e.g., poly(propyleneimine) (PPI) [25], poly(amidoamine) (PAMAM) [26,27,28,29], poly(l-Lysine) (PLL) [30] and carbosilane [31,32] types have been shown to form complexes with siRNA through charge interactions, shielding it from RNases and successfully silencing gene expression in cells making them promising nonviral transfection agents. Unfortunately, all the mentioned dendrimers have high solution stability counteracting an important feature for any carrier i.e., the prerequisite of rapid biodegradation into smaller benign compounds that are safely cleared from the body.

Polyester dendrimers based on 2,2-bis(methylol)propionic acid (bis-MPA) are known for their biocompatibility and the biodegradation of their internal esters [33,34]. In a previous publication, a facile route to postfunctionalization of bis-MPA dendrimers with the amino-acid β-alanine was presented [35]. These dendrimers, featuring primary amino groups as ammonium-trifluoroacetate salts, showed lower cytotoxicity in neurons than PAMAM-analogues and exhibited rapid degradation at physiologically relevant pH and temperature.

In this work, we present the first evaluation of dendrimers based on 2,2-bis(methylol)propionic acid (bis-MPA) as nonviral vectors for siRNA delivery. Dendrimers of generation 1–4 with 6–48 ammonium-trifluoroacetate end-groups are evaluated with respect to siRNA complexation, cytotoxicity, and transfection in cell lines and primary neurons in vitro. Knockdown of mitogen-activated protein kinase 1 (p42-MAPK) is monitored to quantify transfection efficiency. The synthesis of a rhodamine-labeled generation four amino-functional dendron is presented, which allows subcellular localization evaluation of these materials using fluorescence microscopy.

## 2. Results

### 2.1. Synthesis

The synthesis of amino-functional dendrimers of generation 1–4 has been previously published [35]. Here, the same technique was applied to a previously synthesized generation four dendron [36] equipped with a protected tetraethylene glycol linker at its focal point to enable coupling of e.g., fluorophores with less steric hindrance. The amino end-groups were afforded by attachment of boc protected β-alanines through the fluoride-promoted esterification (FPE) protocol [37]. The hydroxyl at the tetraethylene glycol focal point was activated by catalytic hydrogenation, without affecting the boc protections, and rhodamine B was attached through the FPE route. A hydrophobic byproduct suspected to be the lactone form of rhodamine B [38] was observed. This byproduct was easily removed when the boc-protected crude was deprotected with trifluoroacetic acid by extraction from a water solution. The pure amino-functional dendron was then obtained by lyophilization.

**Figure 1:**
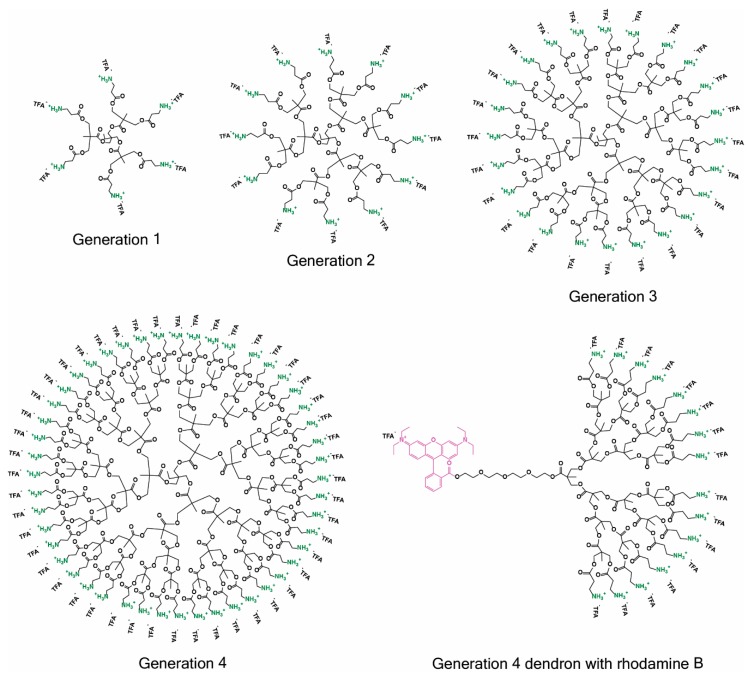
Structures of the generation one to four amino-functional bis-MPA dendrimers that are included in this study, as well as the rhodamine B-labeled generation 4 dendron synthesized to study the subcellular localization of these materials. TFA^-^ is an abbreviation for trifluoroacetate.

All relevant dendritic structures explored in this study are shown in Figure 1. The synthesis of these sophistacted dendritic scaffolds including their analytical data (^1^H-NMR, ^13^C-NMR, MALDI, and fluorescence data for the rhodamine labeled dendron) are presented in detail in the supporting information.

### 2.2. siRNA Complexation and RNase Protection

The complexation ability of the dendrimers to fully complexate all of the siRNA was evaluated by investigating the optimum ratio between the nitrogens on the dendrimer and phosphorous on the siRNA (N/P ratio). This study was performed by agarose gel retardation as specified in the materials & methods section.

All four generations (G1–G4) of TMP-cored amino functional bis-MPA dendrimers were able to complex siRNA, with N/P ratios for complexation ranging from 1.5 to 3 for G2–G4 (data not shown). However, the G1 required a much higher N/P ratio of 19 to fully complex the siRNA. The high N/P ratio found for full complexation may be a due to the previously demonstrated autodegradation of these dendrimers [35] in which loss of amino groups reduces the number of charges on the dendrimer over time. To explore the solution storage stability and the effects of the degradation on their ability to complex the siRNA the dendrimers were stored in a stock solution consisting of a pH 5.5 HEPES buffer at 4 °C and the gel retardations were performed at different time points under the same experimental conditions. Only the G1 showed a significant loss of complexation ability, which is presented in Figure S1 in the supporting information.

**Figure 2:**
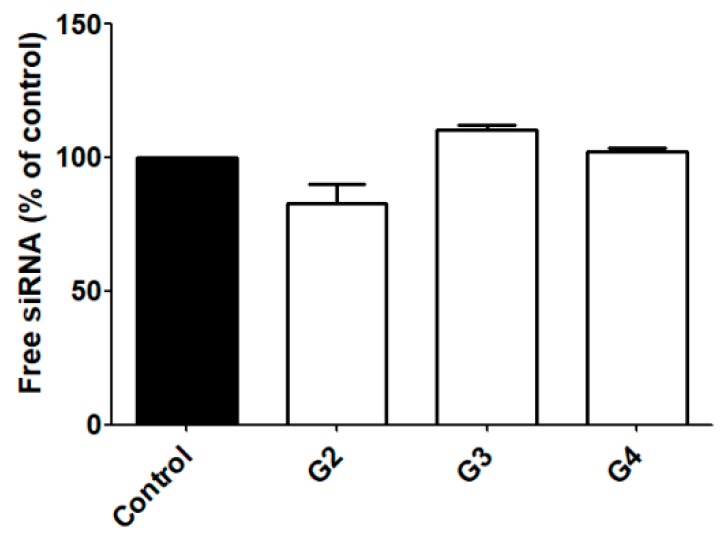
RNase protection. siRNA protection studies for G2–G4 dendrimers were performed at an N/P ratio of 2.5 as indicated in materials & methods. Recovered intact siRNA was measured by densitometric analysis and quantified using Image J. Data represent mean ± s.e.m. of four independent experiments.

Due to these drawbacks, the G1 was excluded from additional studies while the ability of G2–G4 to protect siRNA from RNase-mediated degradation was further explored. As shown in Figure 2, G2–G4 dendrimers were able to protect siRNA from degradation, therefore making them promising candidates as carriers for siRNA transfection.

### 2.3. Cytotoxicity

**Figure 3:**
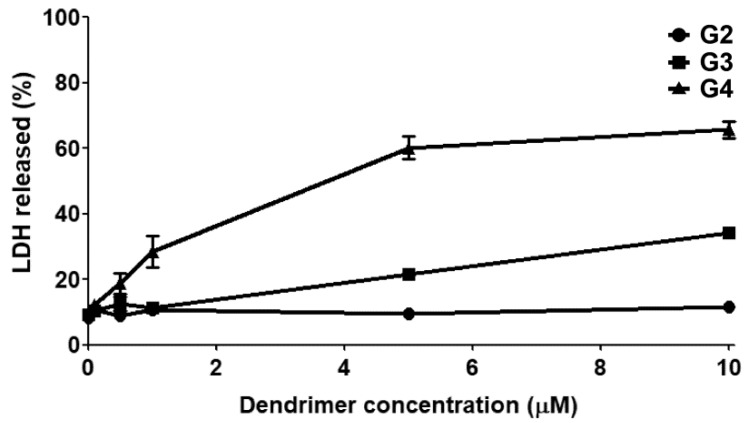
Cytotoxicity in primary neurons. Neurons were exposed to the indicated dendrimer concentration for 72 h. Data are expressed as mean ± s.e.m. of four independent experiments for G2 and G3 and 12 for G4. Where no error bars are visible, they were smaller than the symbol size.

The cytotoxicity of the amino functional bis-MPA dendrimers (G2–G4) was explored in different cell types by monitoring lactate dehydrogenase (LDH) release to the extracellular medium, which is a marker for late irreversible toxicity that indicates cell death. Neither generation showed any toxicity up to 10 µM in rat astrocytes, rat glioma (C6), and human glioblastoma (U87) cell lines (data not shown). The G3 and G4 showed dose dependent toxicity in primary neurons, starting between 1 and 5 μM for G3 and 0.1–1 μM for G4. The G2 showed no signs of toxicity even at the highest tested concentration (10 μM). Figure 3 displays the results from the cytotoxicity experiment on neurons.

### 2.4. Subcellular Localization

**Figure 4:**
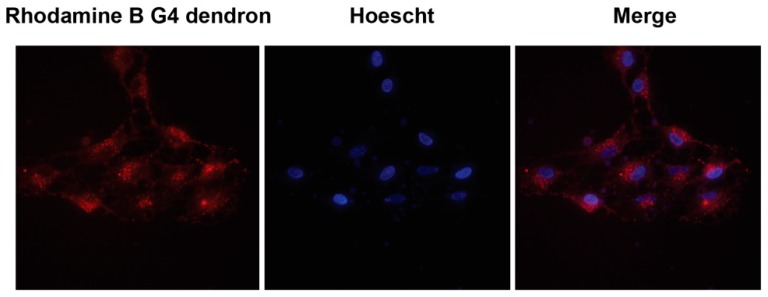
Subcellular tracking of the rhodamine-B labeled G4 dendron. Rat glioma C6 cells were incubated with the G4 dendron (1 μM) for 4 h. Nuclei were labeled with Hoescht 33342. The subcellular distribution of the signal suggests a mitochondrial localization. The experiment shows a representative image of seven different experiments recorded from three different cell cultures.

In order to study the dendrimers ability to enter cells and their localization, C6 and U87 cells were exposed to the rhodamine-labeled G4 dendron for 4 h and studied in a fluorescence microscope. Figure 4 details a representative example of these images with C6 cells, in which the fluorophore is located in mitochondria. 

The stability of the aromatic ester bond between the rhodamine and the dendron was studied using MALDI. No detachment of the rhodamine label was detected for up to 48 h in pH 7.4 and 37 °C even though almost complete loss of β-alanine functional groups and quite extensive loss of internal bis-MPA units were observed. These results are shown in Figures S2–S4.

### 2.5. Transfection

**Figure 5:**
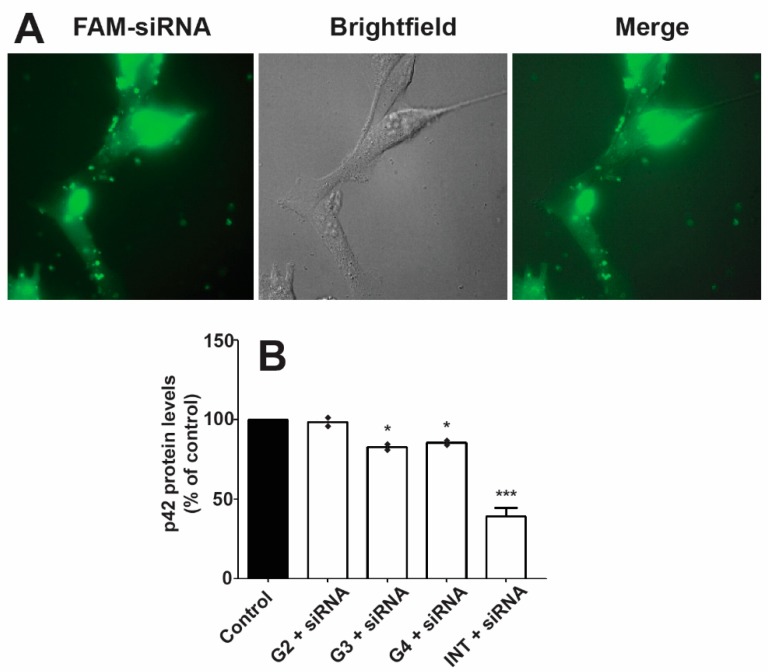
Dendrimer-mediated siRNA transfection. (**A**) Human glioblastoma U87 cells were incubated for 4 h in the presence of complexes (FAM-labeled siRNA (100 nM)/dendrimer (1 μM)) and the image was recorded as indicated in materials & methods. The fluorophore distribution indicates that FAM-siRNA reaches cell cytoplasm. The experiment shows a representative image using the G4 dendrimer from five different experiments recorded from two different cell cultures. (**B**) C6 rat glioma cells were incubated with complexes formed by incubating dendrimers with siRNA (100 nM) designed to degrade p42-MAPK mRNA. Cells were lysed after 72 h and p42-MAPK protein levels were determined. Interferin^®^ (INT) was used as a reference transfection agent. Interferin^®^-mediated scramble siRNA transfection did not show any effect on p42-MAPK protein levels and that data have been omitted from the figure for the sake of clarity. Data represent mean ± s.e.m. of two experiments. * *p* < 0.05; *** *p* < 0.001.

The low toxicity noted for these dendrimers and observed complexation with siRNA make them ideal candidates as transfecting agents. Unfortunately, neither transfection nor protein knockdown of significant levels was observed in primary neurons. However, transfection of carboxyfluorescein(FAM)-labeled siRNA into the cytoplasm was observed in C6 and U87 cells using the G3 and G4 dendrimers. An example image of U87 cells transfected with siRNA using the G4 dendrimer is shown in Figure 5A. A 20% decrease in p42-MAPK protein levels could be detected in rat glioma C6 using the G3 and G4 as siRNA carriers. As a comparison, a decrease of around 60% was observed using the commercially available Interferin^®^ siRNA transfection reagent. The protein knockdown levels are presented in Figure 5B.

## 3. Discussion

### 3.1. siRNA Complexation and RNase Protection

**Figure 6:**
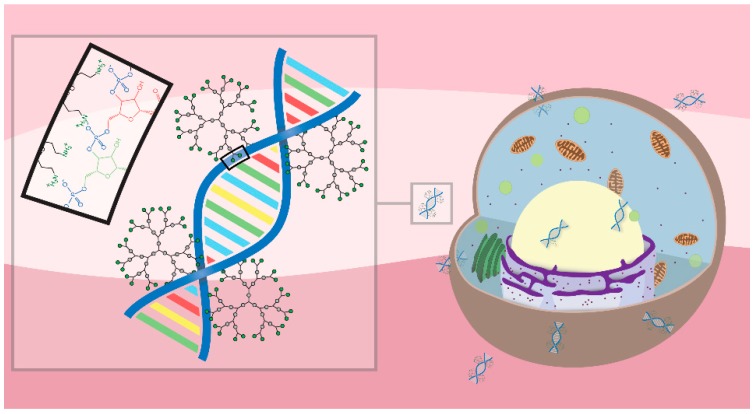
Graphical illustration of the complex formation between amino-functional bis-MPA dendrimers and siRNA, and the transfection of a cell.

The dendrimer–siRNA complexes are formed by charge interactions, where the cationic ammonium groups on the dendrimers interact with the anionic phosphate groups in the siRNA chain, which is illustrated in Figure 6. The required ratio of amino groups on the dendrimer to phosphate groups on the siRNA (N/P ratio) in order bind all of the siRNA in a solution is a measurement of the complexation efficiency. The G1, having only six amino groups able to carry cationic charges as ammonium ions, was very poor at forming complexes with the siRNA requiring a very high excess of amines, while G2–G4 with 12, 24, and 48 terminal amino groups proved very efficient at siRNA complexation with N/P ratios of 1.5:3. This shows the existence of a minimum number of amino groups per dendrimer and/or a minimum molecular weight for efficient siRNA complexation. Similar N/P values for full complexation have been obtained using Tomalia’s PAMAM dendrimers [27,29].

A relevant challenge when using nanoparticles as carriers for therapeutic molecules is the fate of those particles once they have delivered their payload [39]. This issue can be tackled by designing nanoparticles that can be degraded into small molecular fragments that are easily cleared by the target cells [40,41]. Nonetheless, a balance should be maintained between the degradation rate and the time needed to perform their biological/therapeutic actions, and the carriers should have reasonable storage stability. This fact is exemplified by the G1 in Figure S1, for which a reduction in siRNA complexing ability can be observed at different times of storage, caused by the reduction of terminal amino-groups as the dendrimer degrade, which has been previously demonstrated on dendrimers [35] and hereby also on a dendron in Figures S2–S4. However, the loss of function was not observed for higher generation dendrimers, which indicates the existence of a plateau of the complexing capability at a molecular weight and charge somewhere between the G1 and G2, since the complexing ability of G2–G4 was not significantly reduced.

The 21 bp double stranded siRNA used in the experiments can be considered as a rigid stick [42]. This will enable several dendrimers to exist around the rod-like siRNA. The remaining amino/ammonium groups on the dendrimer are thus able to sterically limit the accessibility of the RNase to the nucleic acid and so preventing its degradation, as was observed for G2–G4. A slight degradation of the siRNA of around 20% was observed for the G2, while no significant degradation could be observed for G3 and G4. As a result, it can be concluded that complete protection against RNases is obtained by a theoretical dendrimer between the G2 and G3.

### 3.2. Cytotoxicity

Polycations are known for potential cytotoxicity that is governed by several factors such as the nature of the cationic charge, the structure, and the size/molecular weight [43]. However, PLL based dendrimers, also consisting of amino acids, have shown potential as cationic nanoparticles with low cytotoxicity [44,45]. These dendrimers are composed of amide bonds, which are generally much more stable than the polyester bonds used to build bis-MPA dendrimers. The herein studied dendrimers have similarities to PLL dendrimers, but are more easily degraded into smaller constituents taking advantage of their hydrolytical degradation mechanism. Low cytotoxicity for bis-MPA dendrimers functionalized with lysine, showing the same rapid solution degradation through loss of its terminal lysine groups, has also been reported [46].

None of the studied dendrimers (G1–G4) were cytotoxic in astrocytes, rat glioma (C6), or human glioblastoma (U87) cell lines for up to 10 μM. However, in a previous study [35], the G4 exhibited dose-dependent toxicity at the same concentrations in U87 cells in an MTT assay. The subcellular localization experiments suggested that these materials localize in mitochondria, where the reduction of 3-(4,5-dimethylthiazol-2-yl)-2,5-diphenyltetrazolium bromide (MTT) takes place [47]. One explanation to the discrepancy between the two assays is that the dendrimers, somehow, interfere with the MTT reduction to create false negatives in the assay. Previous studies have shown an increase of reactive oxygen species (ROS) in mitochondria associated with the uptake of cationic polymers [48,49], which has been shown to decrease the activity of MTT reducing enzymes [50]. Moreover, the MTT assay measures early stage reduction in cell viability that eventually leads to cell death, while the LDH assay measures LDH release to the culture medium caused by severe damage to the cellular membrane, which is a generally accepted marker for late stage toxicity. It is possible that the reduction in cell viability, caused by reduction in the mitochondrial functional, is reversible which could be another explanation for this discrepancy.

The G3–G4 showed dose-dependent cytotoxicity on primary rat cortical neurons, while the G2 showed no signs of toxicity towards these cells. This absence of toxicity from the G2 is quite remarkable in itself considering the sensitive nature of primary neurons, and that the G2 possesses twelve primary amino groups per dendrimer. Very similar levels of cell death were previously observed with differentiated neuronal cells cultured with the G4 [35]. In that study, the G2 also proved exceptional biocompatibility in a range of cell lines. The toxicity from the dendrimers most likely arises from the rupture of the cell membranes caused by their cationic exterior. In contrast to G2, the G3 and G4 have a greater number of cationic charges on their exterior, thus making them more efficient at rupturing cell membranes and causing toxicity. The collective results from earlier and current study further cement the influence of the dendritic generation on the cytotoxicity on primary neurons.

### 3.3. Subcellular Localization

Studying the subcellular localization of drug carriers is highly relevant. The carriers should be able to escape the endocytic pathway into relevant organelles and avoid being trapped and destroyed in lysosomes. As exemplified in Figure 4, the fluorophore distribution resembles mitochondrial rather than endosomal distribution. Ester derivatives of rhodamine B (rhodamine 123, 6G, and 3B) have shown to be highly specific fluorescent probes for mitochondria themselves [51]. The uptake of these rhodamine esters is due to their cationic charge at physiological pH and the potential over the inner mitochondrial membrane, resulting in a net negative charge on the inside of the mitochondria [52,53]. Similar mitochondrial localization for the polyester dendrimers, without the rhodamine label, cannot be entirely concluded in this study. However, it is in accordance with the previous reasoning on the discrepancy between the MTT and LDH assays, and numerous cationic species have been shown to selectively localize in mitochondria [54,55,56,57,58]. As for most nanoparticles, the probable cell entrance of the Bis-MPA dendrimers is by endocytosis. This observation is shown in Figure 5A where the diffuse fluorescent pattern indicates endosomal escape. The punctate structures were observed by the presence of fluorescent FAM siRNA in endosomes. Consequently, if the cellular uptake is presumed to be endocytosis, this selective mitochondrial localization suggests a very efficient endo-lysosomal escape [59] of these dendrimers.

### 3.4. Transfection

Efficient transfection should result in a very marked decrease in target protein levels. Unfortunately, the amino functional bis-MPA dendrimers did not cause noticeable transfection of the siRNA into primary neurons at the tested conditions. However, neuronal cells are known to be hard to transfect and are generally sensitive to cytotoxicity.

As shown in Figure 5A, transfection was however observed in U87 cell lines with the G3 and G4 dendrimers, since the fluorescent FAM-labeled siRNA could be seen throughout the cytoplasm. Similar transfection was observed in C6 cells. This transfection resulted in a downregulation of the target protein expression by around 20%. For comparison, the commercial transfection reagent Interferin^®^ was included in the study, with which a 60% downregulation was observed.

A probable reason for this relatively low transfection and protein downregulation could be that the interaction between the dendrimers and the siRNA is not strong enough for the complex to remain intact long enough for the transfection process to occur, or that the dendrimers simply degrade too fast inside the cells, leading to premature release of the siRNA. Another plausible explanation is that the charge-interactions are too strong, inhibiting siRNA release and the formation of the RISC. The dilemma of achieving both strong complexation and efficient dissociation of the siRNA/polymer-complex has been discussed in previous publications [60]. More in-depth studies would be required to determine the cause for this specific system and take measures to improve the protein knockdown levels. However, considering that this is a very initial study of the performance of these dendrimers in delivering siRNA, aspects of the results are promising and give insights into how to construct benign and efficient dendrimers for siRNA delivery [61].

## 4. Materials and Methods

### 4.1. Synthesis Protocols

Amino-functional bis-MPA dendrimers [35], boc-protected β-alanine [35,62], and the tetraethylene glycol bis-MPA dendron [36] were synthesized according to previously published procedures. Other synthesis protocols are presented in the supporting information.

### 4.2. MALDI-ToF-MS

Matrix-assisted laser/desorption ionization time-of-flight mass spectrometry (MALDI-ToF-MS) was performed on a Bruker Ultraflex-III (KTH, Stockholm, Sweden) calibrated with Spherical dendritic calibrants (Polymer Factory; Stockholm, Sweden). Samples were prepared using a mass ratio of 1:1:40 of sample, NaTFA and matrix in tetrahydrofuran (THF). 0.5–1.5 μL of the THF solution was deposited on a stainless steel sample plate using the dried droplet method. *trans*-2-[3-(4-tert-Butylphenyl)-2-methyl-2-propenylidene]malononitrile (DCTB) was used as matrix for nonpolar samples while 2,5-dihydroxybenzoic acid (DHB) was used for polar samples. The acquired spectra were analyzed with FlexAnalysis version 2.2 from Bruker Daltonics (KTH, Stockholm, Sweden).

### 4.3. NMR Spectroscopy

NMR spectroscopy was carried out on a Bruker Avance (KTH, Stockholm, Sweden) III 400 MHz instrument. ^1^H-NMR was carried out using 32 scans, a relaxation delay of 1 s, and a spectral window of 20 ppm. ^13^C-NMR was carried out using 512 scans, a relaxation delay of 2 s, and a spectral window of 240 ppm. Samples were dissolved in chloroform-d or methanol-d (Cambride Isotope Laboratories; Tewksbury, MA, USA). The acquired spectra were analyzed with MestreNova version 9.0.0-12821 from Mestrelab Research (Santiago de Compostela, Spain).

### 4.4. Cell Cultures

Primary cultures of rat cortical neurons were prepared as previously described [63]. The frontolateral cortical lobes were dissected out of Sprague–Dawley embryonic day 17 fetuses, mechanically dissociated and resuspended in serum-free neurobasal medium supplemented with B27 (Invitrogen, Barcelona, Spain) containing 2 mM l-glutamine, penicillin (20 units/mL), and streptomycin (5 μg/mL). The cortical neurons were seeded on PLL-coated culture plates.

Astrocytes were isolated from one day old rat pups as previously described [64] and cultured in Dulbecco’s Modifed Eagle’s Medium (DMEM) (Thermo Fisher; Waltham, MA, USA) supplemented with 10% heat-inactivated fetal calf serum; 2 mM l-glutamine, 5 μg/mL streptomycin, and 20 units/mL penicillin at 37 °C. All animal experimental studies were conducted in accordance with the guidelines of the Ethical Committee of Animal Experimentation at University of Castilla-La Mancha (Albacete, Spain). All studies involving animals are reported in accordance with the guidelines of the European Union (2010/63/EU) for the use of laboratory animals and in accordance with the ARRIVE guidelines for reporting experiments involving animals [65].

C6 rat glioblastoma and U87 human glioblastoma cancer cell lines were obtained from ATCC (Manassas, VA, USA) and were maintained according to the provider instructions. All cell types were maintained at 37 °C in a humidified atmosphere containing 5% CO_2_.

### 4.5. Fluorescence Spectroscopy

Fluorescence spectroscopy of the rhodamine-labeled generation 4 bis-MPA dendron was performed using a Tecan Safire 2 plate reader. Excitation was set to 500 nm and emission was recorded from 280 nm to 800 nm with 5 nm increments.

### 4.6. Fluorescence Microscopy

Fluorescent signals from C6 and U87 cells were recorded as previously described [61]. Cells were seeded at a concentration of 100,000 cells/mL onto 20 mm diameter glass coverslips (Deckglässer; Baden-Württemberg, Germany) and cultured in 6 well plates (Sardstedt; Nümbrecht, Germany) for 24 h in DMEM supplemented with 10% heat-inactivated fetal calf serum; 2 mM l-glutamine, 5 μg/mL streptomycin, and 20 units/mL penicillin maintained at 37 °C in a humidified atmosphere containing 5% CO_2_. Afterwards, cells were treated with the rhodamine-labeled generation 4 bis-MPA dendron (1 µM) in culture medium for 4 h. The cells were then washed three times with Krebs–Henseleit solution with the following ionic composition (in mM): NaCl, 140; CaCl_2_, 2.5; MgCl_2_, 1; KCl, 5; HEPES, 5, Glucose, 11; (pH: 7.4) and mounted on the stage of a Nikon Eclipse TE2000-E fluorescence microscope (Nikon, Tokyo, Japan). The samples containing the cells were stimulated and recorded at the following wavelenghts (excitation/emission): rhodamine B G4 dendron (530 nm/600 nm), FAM-siRNA (488 nm/520 nm), and Hoescht (350 nm/450 nm), and observed through a 40x oil immersion objective. Data was obtained using the NIS Elements AR software (Nikon, Tokyo, Japan).

### 4.7. Agarose Gel Retardation

Agarose gel electrophoresis was performed as previously described [66]. Nanoparticle/siRNA complexes were prepared using 100 nM siRNA with increasing dendrimer concentrations in order to achieve the indicated N/P ratios. The mixture was incubated for 30 min at room temperature and the samples were loaded onto 1.2% agarose gel containing ethidium bromide (50 μg/mL). Electrophoresis was carried on at 60 mV for 15 min, and the resulting gels were photographed under UV illumination. The fluorescent bands were acquired and digitized using a developer (Vilber; Marne La Vallée, France) and analyzed using Image J [67].

### 4.8. siRNA Protection against RNases

Dendrimer-mediated siRNA protection from RNase-mediated degradation was studied as previously described [68]. Nanoparticles, at the indicated concentrations, were incubated for 30 min with siRNA (100 nM) followed by addition of RNase (0.25% *w/v*; Sigma, Barcelona; Spain) and incubation for another 30 min at 37 °C. RNase was then inactivated by keeping the samples at 4 °C for 15 min and after which heparin (0.5 USP units) was added. The samples were kept at 4 °C for an additional 20 min to completely release siRNA from the nanoparticles while RNase remained inactivated as previously described [29]. Samples were then loaded onto a 1.2% agarose gel containing ethidium bromide (50 μg/mL), and run under the same experimental conditions as indicated above. Fluorescent bands were acquired and digitized using a developer (Vilber, Marne-la-Vallée, France) and analyzed using the Image J software [67].

### 4.9. siRNA Transfection & Western Blot Analysis

Cells were incubated either with the dendrimer alone or with the dendrimer/siRNA dendriplexes formed by incubating the corresponding dendrimer (0.5 μM) with either scramble noncoding siRNA or specific siRNA (100 nM; Sigma, Barcelona, Spain) for rat p42-MAPK (sense: 5′-GUAUAUACAUUCAGCUAAUAU-3′, antisense: 5′-AUAUUAGCUGAAUGUAUAUAC-3′) for 30 min as previously described [61]. Cells were treated for 72 h, the medium was washed twice and the cells were lysed. Western blots were performed as previously described using 15% SDS-PAGE gels [69]. Polyclonal anti-p42-MAPK antibody (1:1000) (Cell Signaling Technology; Danvers, MA, USA) and polyclonal anti-β-actin antibody (1:4000) (Sigma Chemical Co.; St. Louis, MO, USA) were used to correct for protein loading. Immunocomplexes were visualized using an enhanced chemiluminescence system (Millipore, Burlington, MA, USA). For comparison, the commercial Interferin^®^ transfection reagent was included in the study and used according to its specified protocol. Transfection using FAM-labeled siRNA was performed using previously published protocol [61]. Densitometric analysis of immunoreactive bands was performed using Image J [67].

### 4.10. Cytotoxicity Studies

For viability experiments, LDH release to the incubation medium was used as an index of cellular death as previously described [70]. Cells were cultured in 24-well culture plates at a concentration of 100,000 cells/mL and treated with different dendrimer concentrations ranging from 0.1 to 10 µM for 72 h in culture medium. The culture medium was collected, the cells were washed with phosphate-buffered saline, and lysed using 0.9% Triton X-100 (*v/v*) in saline. The LDH activity present in the culture media, as well as that present in the lysates, was measured spectrophotometrically at 490 nm on a 96-well plate reader using the Cytotox 96 Kit (Promega; Madrid, Spain). Percentage of toxicity was calculated as the percentage of LDH released in comparison to the LDH present in the cells at the beginning of the experiment as previously described [70].

### 4.11. Degradation

The generation four rhodamine-labeled amino-functional dendron was dissolved in a McIlvaine buffer of pH 7.4 at a concentration of 0.5 mM. The pH of the solution was reconfirmed after the addition of the dendron. At set time points, 3 μL aliquots were mixed with 20 μL of a solution with 1 g/L of NaTFA and 10 g/L of 2,5-DHB. 1 μL of this solution was deposited on a stainless steel MALDI-plate and analyzed with MALDI-ToF-MS.

## 5. Conclusions

Amino-functional bis-MPA dendrimers of generation one to four have been investigated as nonviral vectors for siRNA delivery in primary neurons, astrocytes, glioma, and glioblastoma cell lines. The G2–G4 dendrimers were efficient at forming charge-interaction complexes with siRNA at N/P ratios of 1.5:3 for full siRNA complexation, and they were nontoxic to astrocytes, glioma, and glioblastoma cell lines. G3-G4 showed dose-dependent cytotoxicity towards primary neurons, while the G2 showed no signs of toxicity at the tested concentrations. Fluorescence microscopy experiments suggested efficient endo-lysosomal escape by the dendrimers, with distinct mitochondrial subcellular localization. Transfection and protein downregulation was not observed in primary neurons. However, transfection was successful into rat glioma and human glioblastoma cells, followed by a slight reduction in target protein expression of around 20%. This is the first reported evaluation of bis-MPA dendrimers for the intracellular delivery of siRNA, where the benefits and challenges of these materials are outlined.

## Supplementary Materials

The following are available online, Figure S1: Solution storage influence on the siRNA complexation of G1. Figures S2–S4: Degradation of the fluorescent G4 dendron. Figure S5: Fluorescence spectrum of the rhodamine B G4 dendron. Synthesis protocols, NMR- and MALDI-spectra.
