# Supplementary material for: Evaluation of Amino-Functional Polyester Dendrimers Based on Bis-MPA as Nonviral Vectors for siRNA Delivery
Source: Molecules. 2018 Aug 14;23(8):2028. doi: 10.3390/molecules23082028 (PMC6222295; doi:10.3390/molecules23082028)
Supplement: Supplementary file 1 [file molecules-23-02028-s001.pdf]

molecules

Supporting information to "Amino-functional polyester dendrimers based on bis-MPA as non-viral vectors for siRNA delivery"

P. Stenström, D. Manzanares, Y. Zhang, V. Ceña and M. Malkoch*
* To whom correspondence should be addressed (malkoch@kth.se)

Table of Contents

MATERIALS FOR SYNTHESIS AND CHARACTERIZATION 2

SYNTHESIS PROTOCOLS 2

SYNTHESIS OF BNTEG-G4-OH 2

SYNTHESIS OF BNTEG-G4-NHBOC 3

SYNTHESIS OF OH-TEG-G4-NHBOC 4

SYNTHESIS OF RHB-TEG-G4-NHBOC 5

SYNTHESIS OF RHB-TEG-G4-$NH_3^+TFA^-$ 6

DEGRADATION 7

SOLUTION STORAGE INFLUENCE ON THE SIRNA COMPLEXATION OF TMP-G1-$NH_3^+TFA^-$ 7

DEGRADATION CHARACTERISTICS OF RHB-TEG-G4-$NH_3^+TFA^-$ 7

FLUORESCENCE INTENSITY MEASUREMENT 9

FLUORESCENCE OF RHB-TEG-G4-$NH_3^+TFA^-$ 9

NMR-SPECTRA 9

^{1}H NMR (400 MHZ, METHANOL-D4) BNTEG-G4-OH 9

^{13}C NMR (101 MHZ, METHANOL-D4) BNTEG-G4-OH 10

^{1}H NMR (400 MHZ, CHLOROFORM-D) BNTEG-G4-NHBOC 10

^{13}C NMR (101 MHZ, CHLOROFORM-D) BNTEG-G4-NHBOC 11

^{1}H NMR (400 MHZ, CHLOROFORM-D OH-TEG-G4-NHBOC 11

^{13}C NMR (101 MHZ, CHLOROFORM-D) OH-TEG-G4-NHBOC 12

^{1}H NMR (400 MHZ, CHLOROFORM-D) RHB-TEG-G4-NHBOC 12

^{13}C NMR (101 MHZ, CHLOROFORM-D) RHB-TEG-G4-NHBOC 13

^{1}H NMR (400 MHZ, METHANOL-D4) RHB-TEG-G4-$NH_3^+TFA^-$ 13

^{13}C NMR (101 MHZ, METHANOL-D4) RHB-TEG-G4-$NH_3^+TFA^-$ 14

MALDI-SPECTRA 14

BnTEG-G4-OH 14
BnTEG-G4-NHBoc 15
OH-TEG-G4-NHBoc 15
RhB-TEG-G4-NHBoc 15
RhB-TEG-G4-$NH_3^+TFA^-$ 16

Materials for synthesis and characterization

Ethyl acetate (EtOAc), dichloromethane (DCM) and tetrahydrofuran (THF) were purchased from Merck. Chloroform and dimethyl formamide (DMF) were purchased from VWR. Rhodamine B, Cesium fluoride (CsF), sodium trifluoroacetate (NaTFA), *trans*-2-[3-(4-tert-Butylphenyl)-2-methyl-2-propenylidene]malononitrile (DCTB) and 2,5-dihydroxybenzoic acid (DHB), Palladium on carbon (5 wt.% loaded) were purchased from Sigma Aldrich. Hydrogen gas was purchased from Strandmøllen. β-alanine and DOWEX 50WX8 was purchased from Alfa Aesar. N,N'-carbonyl diimidazole (CDI) was purchased by Carbosynth. Deuterated solvents were purchased from Cambridge Isotope Laboratories. SpheriCal dendritic calibrants for MALDI-MS were obtained in kind from Polymer Factory, Stockholm, Sweden.

Synthesis protocols

Synthesis of BnTEG-G4-OH

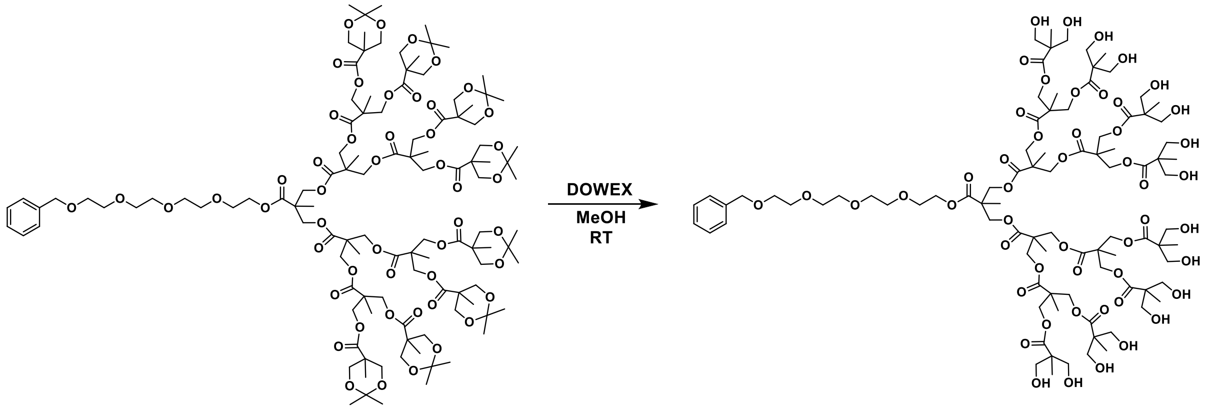

1.2 g (511 μmol, 1 eq) of BnTEG-G4-Acetonide was dissolved in methanol. 300 mg of DOWEX 50WX8 was added, and the mixture was allowed to stir at room temperature. After 3h and 6h the mixture was filtered and 300 mg of new DOWEX 50WX8 was added. After the exchange at 6h, the mixture was stirred with DOWEX 50WX8 for 14h at room temperature. Complete conversion of the acetonide groups into diols was confirmed with MALDI-TOF MS. The DOWEX 50WX8 was filtered off and the methanol was evaporated to give 0.918 g (453 μmol, 0.89 eq) of pure product as a white sticky solid.
^{1}H NMR (400 MHz, *Methanol-d$_4$*) δ 7.46 – 7.21 (m, 5H, **Ar**-CH_2), 4.56 (s, 2H, Ar-C**H$_2$**-O), 4.40 – 4.20 (m, 30H, C-C**H$_2$**-O), 3.73 (t, J = 5.9, 3.6 Hz, 2H, O-C**H$_2$**-CH_2), 3.72 – 3.56 (m,

42H, O-C**H**$_2$-C**H**$_2$-O, C-C**H**$_2$-O), 1.33 (s, 3H C-C**H**$_3$), 1.30 (2·s, J = 2.7 Hz, 17H, C-C**H**$_3$), 1.15 (s, 24H, C-C**H**$_3$).

^{13}C NMR (101 MHz, *Methanol-d$_4$*) δ 175.83 (**C**=O), 173.74 (**C**=O), 173.69 (**C**=O), 173.19 (**C**=O), 139.56 (**Ar**-CH$_2$), 129.35 (**Ar**-CH$_2$), 128.88 (**Ar**-CH$_2$), 128.67 (**Ar**-CH$_2$), 74.07 (Ar-**C**H$_2$-O), 71.52 (O-CH$_2$-**C**H$_2$-O), 71.49 (O-**C**H$_2$-CH$_2$-O), 71.41 (O-**C**H$_2$-**C**H$_2$-O), 70.56 (O-**C**H$_2$-**C**H$_2$-O), 69.82 (O-**C**H$_2$-CH$_2$), 67.44 (CH$_2$-**C**H$_2$-O), 66.99 (C-**C**H$_2$-O), 66.08 (C-**C**H$_2$-O), 65.77 (C-**C**H$_2$-O), 65.67 (C-**C**H$_2$-O), 51.70 (**C$_{quaternary}$**), 47.99 (**C$_{quaternary}$**), 47.85 (**C$_{quaternary}$**), 18.27 (C-**C**H$_3$), 18.09 (C-**C**H$_3$), 18.06 (C-**C**H$_3$), 17.32 (C-**C**H$_3$).

MALDI: $(M+Na^+)_{calculated}$: 2047.86 Da $(M+Na^+)_{found}$: 2054.28 Da (100.31% of calculated)

Synthesis of BnTEG-G4-NHBoc

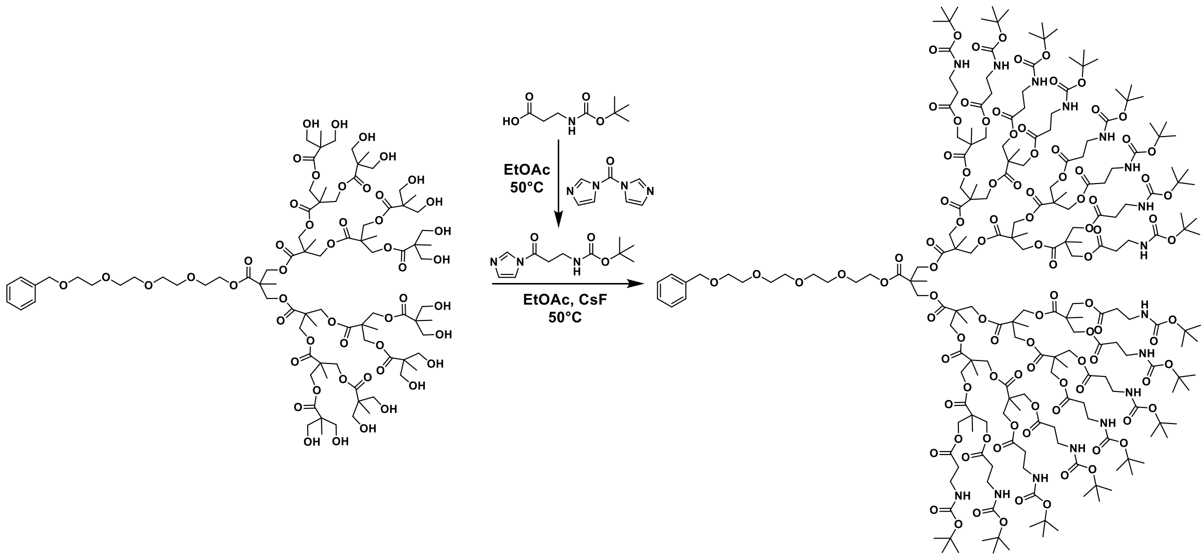

985 mg (5.21 mmol, 24 eq) of boc protected β-alanine was dissolved in 5 mL of EtOAc at 50°C. 928 mg (5.73 mmol, 26.4 eq) of CDI was added. The imidazole-activation of the β-alanine was carried out for 30 minutes under nitrogen, and full conversion was confirmed with ^{1}H-NMR. 440 mg (217 μmol, 1 eq) of BnTEG-G4-OH was added together with 105 mg (694 μmol, 3.2 eq) of CsF. The reaction was carried out for 14h at 50°C under nitrogen and full conversion was confirmed with MALDI-TOF MS. The excess of imidazole-activated β-alanine was quenched by adding 1 mL of deionized water and stirring the mixture at room temperature for 6h. The organic phase was diluted with 60 mL of EtOAc, and washed with 4x20 mL of 10% $NaHSO_4$ in H_2O, 4x20 mL of 10% $NaHCO_3$ in H_2O and 20 mL of brine. The organic phase was dried with $MgSO_4$, filtered and rotoevaporated to give 803 mg (169 μmol, 0.80 eq) of pure product as a slightly yellow sticky solid.

^{1}H NMR (400 MHz, *Chloroform-d*) δ 7.36 – 7.25 (m, 5H, **Ar**-CH$_2$), 5.28 (s, 16H, CH$_2$-N**H**-Boc), 4.55 (s, 2H, Ar-C**H**$_2$-O), 4.40 – 4.09 (m, 65H, C-C**H**$_2$-O), 3.73 – 3.58 (m, 16H, O-C**H**$_2$-C**H**$_2$-O), 3.36 (q, J = 6.1 Hz, 32H, CH$_2$-C**H**$_2$-NH), 2.54 (t, J = 6.1 Hz, 31H, C**H**$_2$-CH$_2$-NH), 1.43 (s, 144H, NH-**Boc**), 1.33 – 1.20 (m, 45H, C-C**H**$_3$).

^{13}C NMR (101 MHz, *Chloroform-d*) δ 172.01 (**C**=O), 171.94 (C=O) 171.83 (**C**=O), 171.51 (**C**=O), 171.38 (**EtOAc**), 171.14 (**C**=O), 155.86 (**C**=O (Boc)), 138.25 (**Ar**-CH$_2$), 128.39 (**Ar**-CH$_2$), 127.74 (**Ar**-CH$_2$), 127.65 (**Ar**-CH$_2$), 79.31 (Ar-**C**H$_2$-O), 73.22 (O-**C**H$_2$-**C**H$_2$-O), 70.63

(O-**C**H2-**C**H2-O), 70.56 (O-**C**H2-**C**H2-O), 70.41 (O-**C**H2-**C**H2-O), 69.43 (O-**C**H2-**C**H2-O), 68.74 (O-CH2-**C**H2-O), 65.67 (C-**C**H2-O), 65.12 (C-**C**H2-O), 64.80 (C-**C**H2-O), 64.50 (C-**C**H2-O), 60.40 (**EtOAc**), 46.68 (**C**$_{\textbf{quaternary}}$), 46.45 (**C**$_{\textbf{quaternary}}$), 36.15 (CH2-**C**H2-NH), 34.45 (**C**H2-CH2-NH), 28.44 (C-**C**H3 (Boc)), 21.07 (**EtOAc**), 17.87 (C-**C**H3), 17.57 (C-**C**H3), 17.50 (C-**C**H3), 17.44 (C-**C**H3), 14.24 (**EtOAc**).

MALDI: $(M+Na^+)_{calculated}$: 4785.29 Da $(M+Na^+)_{found}$: 4788.80 Da (100.07% of calculated)

Synthesis of OH-TEG-G4-NHBoc

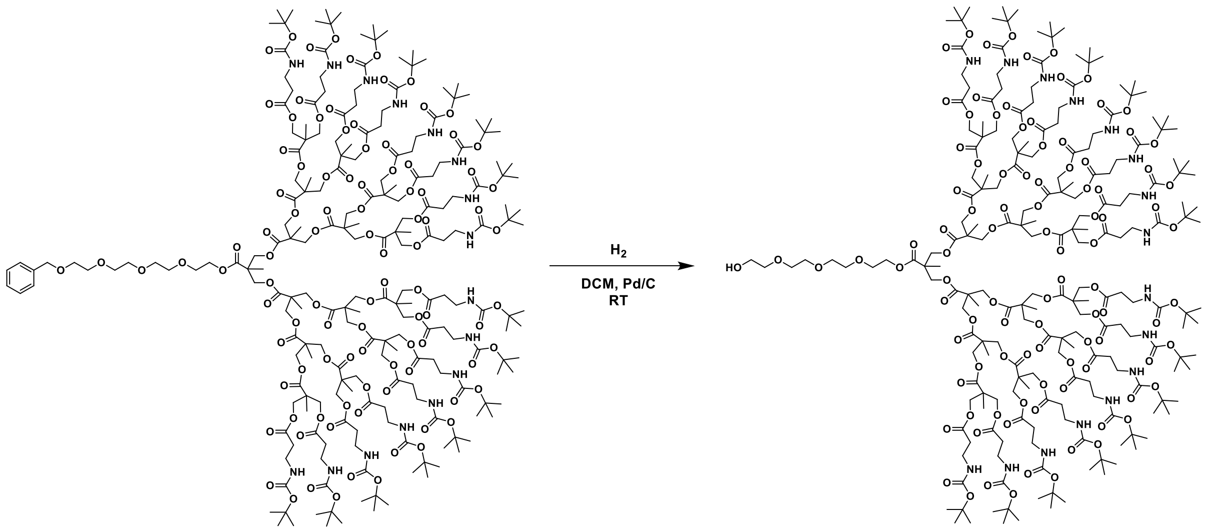

28 mg (10 wt.%) of 5 wt.% palladium loaded on activated carbon was suspended in 10 mL of DCM. The suspension was added to a 100 mL round-bottomed flask with 280 mg (58.8 µmol, 1 eq) of BnTEG-G4-NHBoc. Another 10 mL of DCM was added, and the flask was fitted with a rubber septum and back-filled with hydrogen gas from a soccer ball through a needle. 2 mL of methanol was added through a needle and the mixture was stirred for 8h at room temperature. Full conversion was confirmed with MALDI-TOF MS, and the mixture was filtered through a P5 glass filter. The solvent was evaporated to give 245 mg (52.5 µmol, 0.89 eq) of pure product as a white sticky solid.

^{1}H NMR (400 MHz, *Chloroform-d*) δ 5.27 (s, 14H, CH2-N**H**-Boc), 4.41 – 4.06 (m, 62H C-C**H2**-O), 3.74 – 3.55 (m, 15H, O-C**H2**-C**H2**-), 3.35 (q, J = 6.1 Hz, 32H, CH2-C**H2**-NH), 2.53 (t, J = 6.1 Hz, 31H, C**H2**-CH2-NH), 1.42 (s, 144H, NH-**Boc**), 1.32 – 1.19 (m, 47H, C-C**H3**).

^{13}C NMR (101 MHz, *Chloroform-d*) δ 172.10 (**C**=O), 171.95 (**C**=O), 171.61 (**C**=O), 171.47 (**C**=O), 155.96 (**C**=O (Boc)), 79.43 (**C**$_{\textbf{quaternary}}$ (Boc)), 72.63 (**C**H2-**C**H2-O), 70.67 (**C**H2-**C**H2-O), 70.62 (**C**H2-**C**H2-O), 70.51 (**C**H2-**C**H2-O), 70.43 (**C**H2-**C**H2-O), 65.21 (C-**C**H2-O), 64.90 (C-**C**H2-O), 64.61 (C-**C**H2-O), 61.70 (C-**C**H2-O), 46.77 (**C**$_{\textbf{quaternary}}$), 46.54 (**C**$_{\textbf{quaternary}}$), 36.23 (CH2-**C**H2-NH), 34.55 (**C**H2-CH2-NH), 28.53 (C-**C**H3 (Boc), 17.96 (C-**C**H3), 17.66 (C-**C**H3), 17.58 (C-**C**H3).

MALDI: $(M+Na^+)_{calculated}$: 4695.25 Da $(M+Na^+)_{found}$: 4696.92 Da (100.04% of calculated)

Synthesis of RhB-TEG-G4-NHBoc

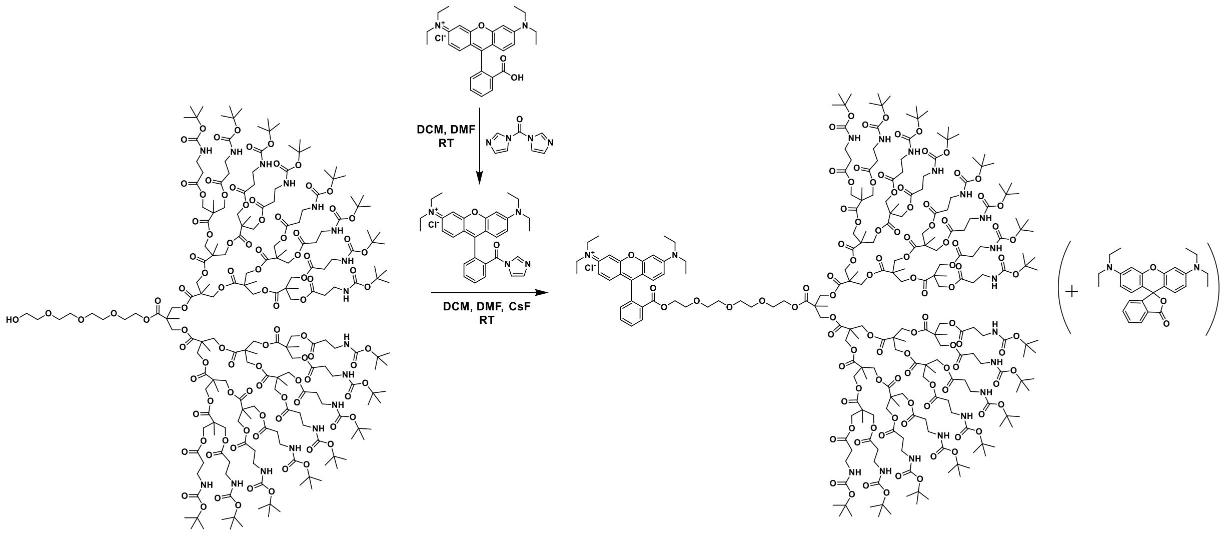

16 mg (33.4 µmol, 1.6 eq) of rhodamine B was dissolved in 0.5 mL of a mixture of 1:1 chloroform and DMF. 5.4 mg (33.4 µmol, 1.6 eq) of CDI was added. The formation of imidazole-activated rhodamine B was carried out for 1h at room temperature. The solution was added to 100 mg (21.6 µmol, 1 eq) of OH-TEG-G4-NHBoc together with 1.0 mg (6.6 µmol, 0.3 eq) of CsF. The reaction was carried out for 14h at room temperature and full conversion was confirmed by MALDI-TOF MS. The excess of imidazole-activated rhodamine B was quenched by the addition of 100 µL of water and stirring for 6h. The reaction mixture was diluted with 50 mL of chloroform and washed with 4x20 mL of 10% $NaHSO_4$ in H_2O, 4x20 mL of 10% $NaHCO_3$ in H_2O and 20 mL of brine. The organic phase was dried with $MgSO_4$, filtered and rotoevaporated to give 108 mg (21.2 µmol, 0.98 eq. crude) of product as a dark red/magenta sticky solid containing the hydrophobic lactone form of rhodamine B as an impurity that was removed in the next reaction step where the boc-protective groups are removed.

^{1}H NMR (400 MHz, *Chloroform-d*) δ 8.33 (d, J = 8.0 Hz, 1H, CH=C**H**-C(C=O)), 8.09* (d, J = 7.4 Hz, 3H), 7.78 (dt, J = 30.9, 8.7 Hz, 3H, CH-C**H**=C**H**-CH), 7.61* (dt, J = 17.4, 7.3 Hz, 7H), 7.29 (d, J = 7.6 Hz, 1H, C=C**H**-CH), 7.19* (d, J = 7.3 Hz, 4H), 7.06 (d, J = 9.5 Hz, 2H, C-C**H**=C**H**-C(NEt$_2$)), 6.94 (d, J = 10.1 Hz, 2H, C-C**H**=C**H**-C(NEt$_2$)), 6.82 (s, 2H, C(NEt$_2$)-C**H**=C), 6.71* (d, J = 9.0 Hz, 7H), 6.50* (s, 7H), 6.44* (d, J = 9.1 Hz, 7H), 5.30 (s, 16H, N**H**Boc), 4.33 – 4.09 (m, 65H, C-C**H$_2$**-O), 3.65 (m, 16H, C-C**H$_2$**-O), 3.41* (q, J = 7.1 Hz, 28H), 3.36 (q, J = 6.5 Hz, 36H, CH$_2$-C**H$_2$**-NH), 2.53 (t, J = 6.2 Hz, 32H, C**H$_2$**-CH$_2$-NH), 1.42 (s, 144H, NH**Boc**), 1.24 (m, C-C**H$_3$**), 1.20* (t, 39H).

13C NMR (101 MHz, *Chloroform-d*) δ 172.11 (**C**=O), 171.95 (**C**=O), 155.97 (**C**=O (Boc)), 129.92*, 129.51*, 109.72*, 97.28*, 79.42 (**C$_{quaternary}$** (Boc)), 64.89 (C-**C**H$_2$-O), 46.77 (**C$_{quaternary}$**), 46.54 (**C$_{quaternary}$**), 46.31 (**C$_{quaternary}$**), 45.01*, 36.24 (CH$_2$-**C**H$_2$-NH), 34.54 (**C**H$_2$-CH$_2$-NH), 33.97, 29.83, 28.53 (C-**C**H$_3$ (Boc), 25.78, 25.08, 17.97 (C-**C**H$_3$), 17.67 (C-**C**H$_3$), 12.69*.

*Peaks from impurity suspected to be lactone rhodamine B.

MALDI: $(M+H^+)_{calculated}$: 5099.50 Da $(M+Na^+)_{found}$: 5103.46 Da (100.08% of calculated)

Synthesis of RhB-TEG-G4-$NH_3^+TFA^-$

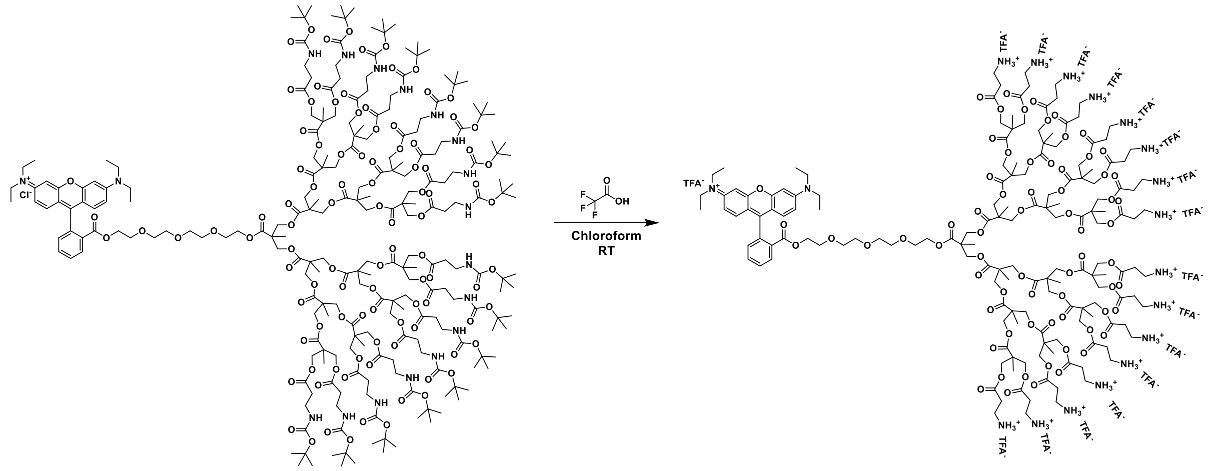

100 mg (19.6 µmol, 1 eq crude) of RhB-TEG-G4-NHBoc containing ring-closed rhodamine B was dissolved in 1 mL of a 1:1 mixture of trifluoroacetic acid and chloroform. The reaction was carried out for 1.5h at room temperature. Full conversion was confirmed with MALDI-TOF MS, where the dendron is detected as a freebase due to the instrument being calibrated for and operated in negative mode, where the trifluoroacetate counter-ions are not able to move through the TOF chamber. After evaporation of the TFA and chloroform, the product was redissolved in 3 mL of deionized water, and the aqueous phase was treated 4 times with 1 mL of chloroform to remove the ring-closed rhodamine b formed in the previous reaction step. 70 mg (12.9 µmol, 0.66 eq relative to crude) of pure product was obtained as a dark red/magenta sticky solid after lyophilization of the aqueous phase.

^{1}H NMR (400 MHz, *Methanol-d4*) δ 8.35 (d, J = 7.9 Hz, 1H, **Rhodamine B-**), 8.08 (s, 2H, **Rhodamine B-**), 7.85 (dt, J = 24.7, 7.7 Hz, 2H, **Rhodamine B-**), 7.45 (d, J = 7.8 Hz, 1H, **Rhodamine B-**), 7.18 – 7.01 (m, 3H, **Rhodamine B-**), 7.00 (s, 1H, **Rhodamine B-**), 4.41 – 4.22 (m, 60H), 3.59 (m, 21H, O-CH_2-**C**H_2-O, C**H**$_2$-CH_3 (Rhodamine B)), 3.25 (t, J = 6.9 Hz, 33H), 2.83 (t, J = 6.7 Hz, 32H), 1.40 – 1.24 (m, 57H, C-C**H**$_3$, CH_2-C**H**$_3$ (Rhodamine B)).

^{13}C NMR (101 MHz, *Methanol-d4*) δ 173.53 (**C**=O), 173.30 (**C**=O), 173.19 (**C**=O), 171.86 (**C**=O), 162.52 (**Rhodamine B-**), 162.16 (**Rhodamine B-**), 132.41 (**Rhodamine B-**), 115.46 (**Rhodamine B-**), 114.84 (**Rhodamine B-**), 97.25 (**Rhodamine B-**), 71.34 (O-**C**H_2-**C**H_2-O), 66.83 (C-**C**H_2-O), 66.52 (C-**C**H_2-O), 48.00 (**C$_{quaternary}$**), 47.63 (**C$_{quaternary}$**), 46.84 (**C**H_2-CH_3 (Rhodamine B)), 36.32 (**C**H_2-CH_2-NH), 32.20 (C-**C**H_3 (Boc), 18.20 (C-**C**H_3), 18.10 (C-**C**H_3), 12.87 (CH_2-**C**H_3 (Rhodamine B)).

MALDI: $(M+H^+)_{calculated}$: 3498.66 Da $(M+H^+)_{found}$: 3498.60 Da (100.00% of calculated)

Degradation

Solution storage influence on the siRNA complexation of TMP-G1-$NH_3^+TFA^-$

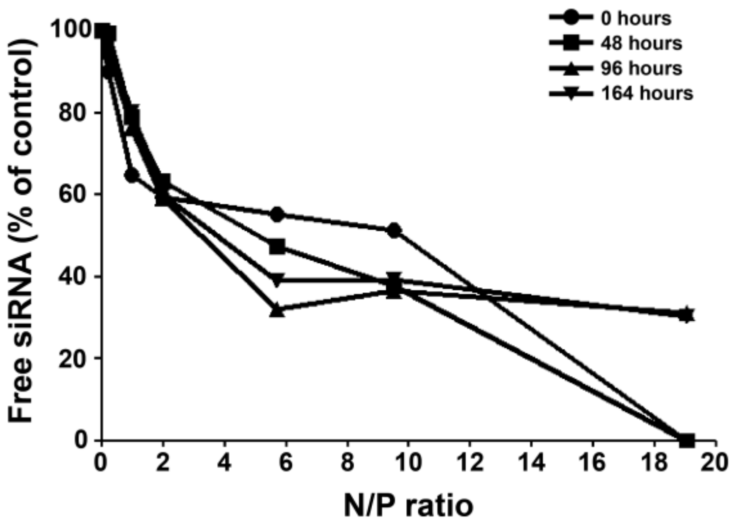

Figure S1. Solution storage influence on the siRNA complexation of G1. siRNA (100 nM) was incubated with the first generation dendrimer at different N/P ratios and loaded onto a 1.2% agarose gel as indicated in materials & methods. The experiment was repeated after storing the dendrimer at 4°C in a pH 5.5 HEPES buffer for the indicated times.

Degradation characteristics of RhB-TEG-G4-$NH_3^+TFA^-$

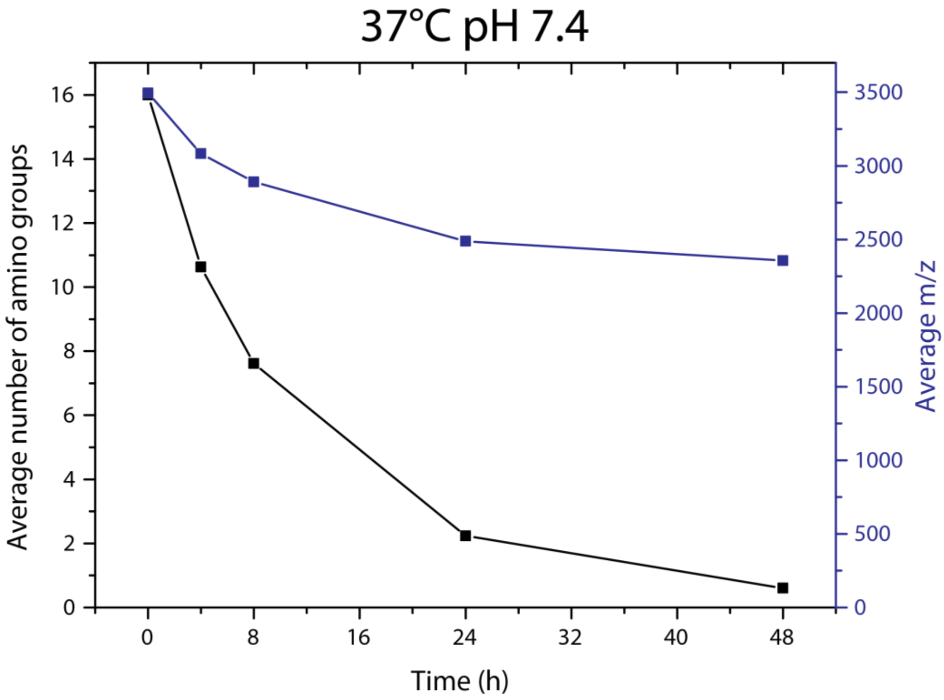

Figure S2. Average number of amino groups and m/z observed in MALDI-ToF-MS for of RhB-TEG-G4-$NH_3^+TFA^-$ after 4, 8, 24 and 48 hours in pH 7.4.

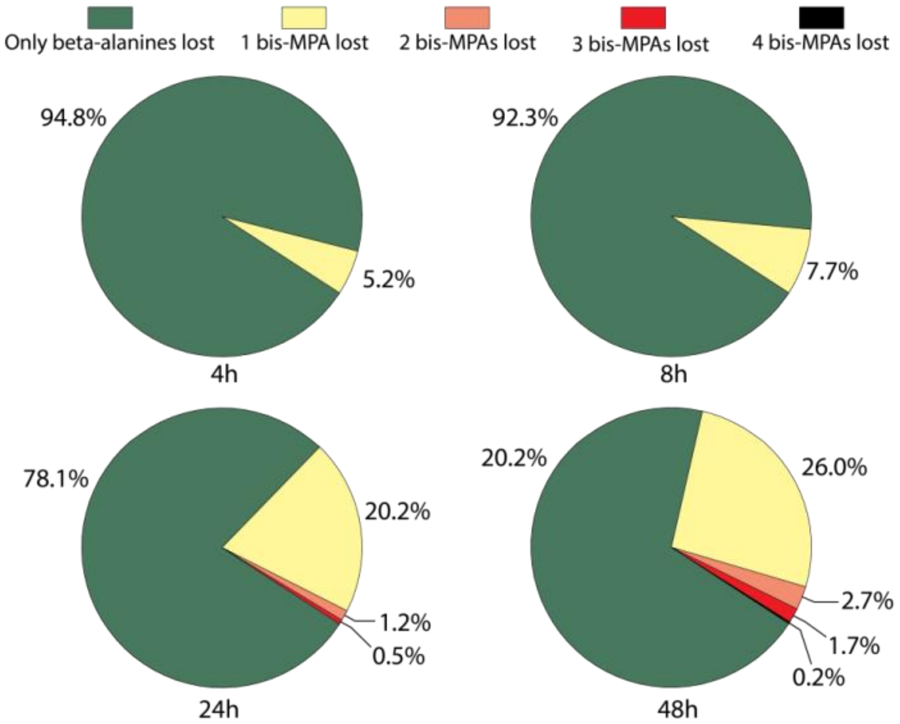

Figure S3. Charts describing the characteristics of the degradation of RhB-TEG-G4-$NH_3^+TFA^-$ after 4, 8, 24 and 48 hours in pH 7.4.

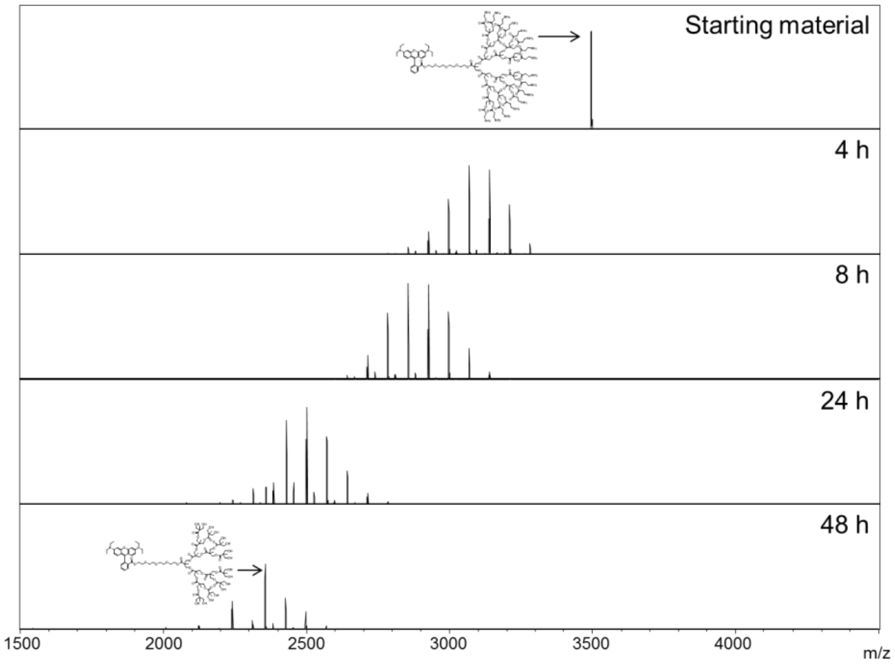

Figure S4. MALDI-spectra acquired during the degradation experiment.

Fluorescence intensity measurement

Fluorescence of RhB-TEG-G4-$NH_3^+TFA^-$

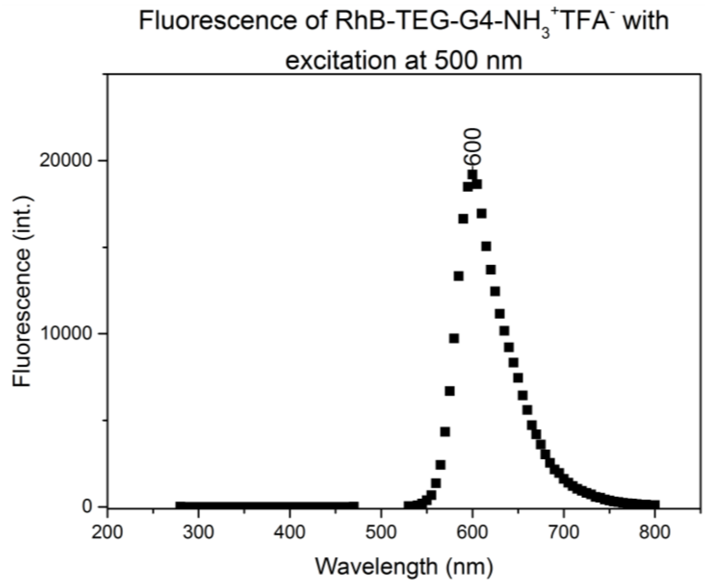

Figure S5. Fluorescence intensity of RhB-TEG-G4-$NH_3^+TFA^-$ at various wavelengths with excitation at 500 nm.

NMR-spectra

^{1}H NMR (400 MHz, Methanol-d_4) BnTEG-G4-OH

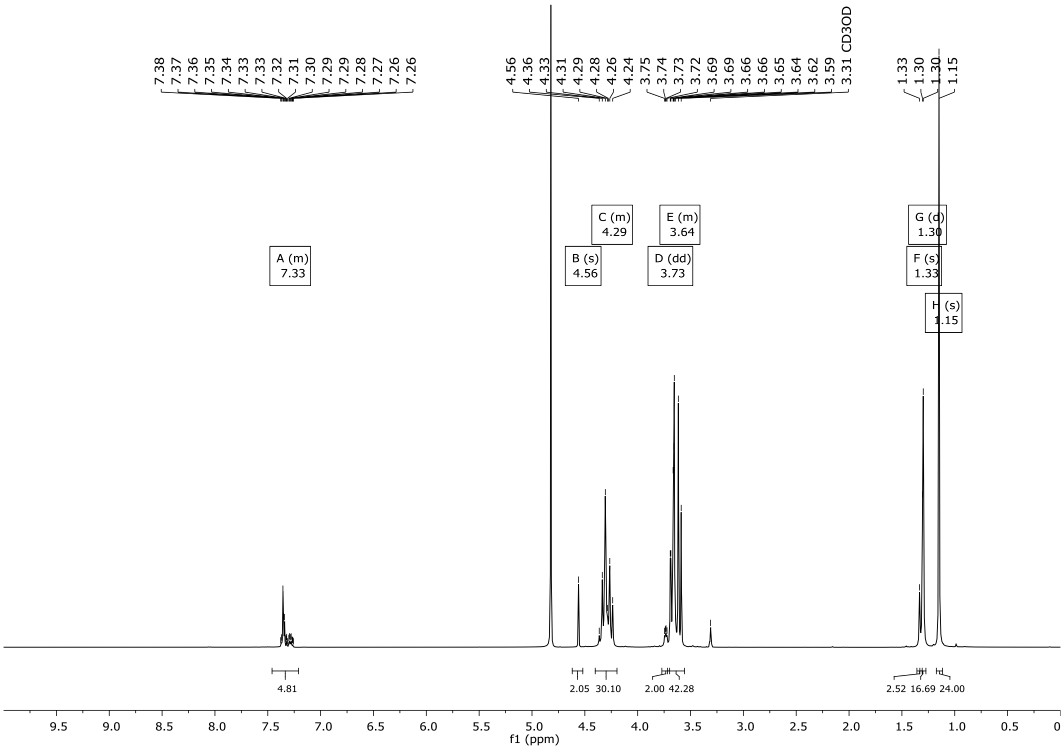

^{13}C NMR (101 MHz, Methanol-d$_4$) BnTEG-G4-OH

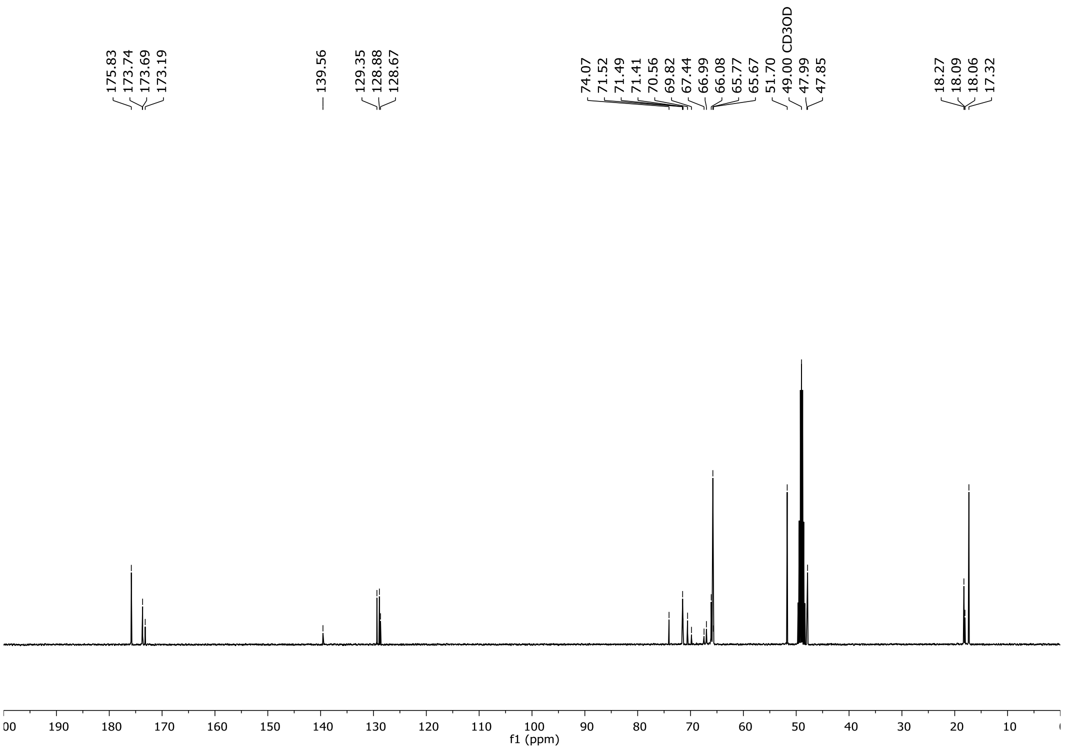

^{1}H NMR (400 MHz, Chloroform-d) BnTEG-G4-NHBoc

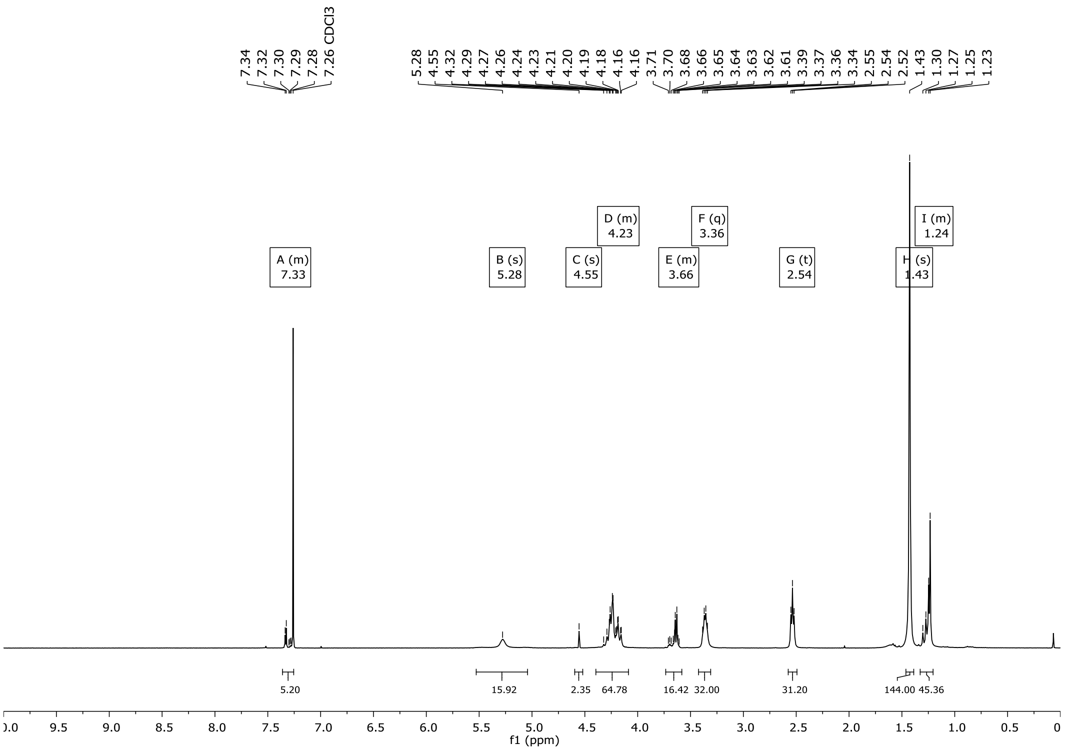

^{13}C NMR (101 MHz, Chloroform-d) BnTEG-G4-NHBoc

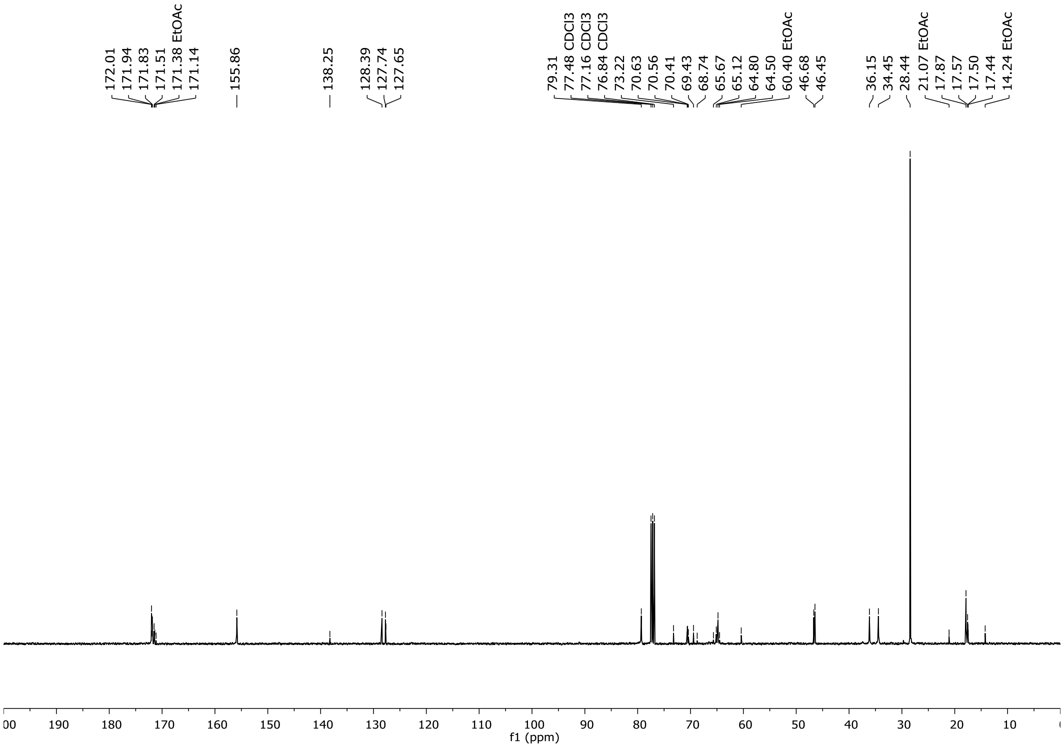

^{1}H NMR (400 MHz, Chloroform-d OH-TEG-G4-NHBoc

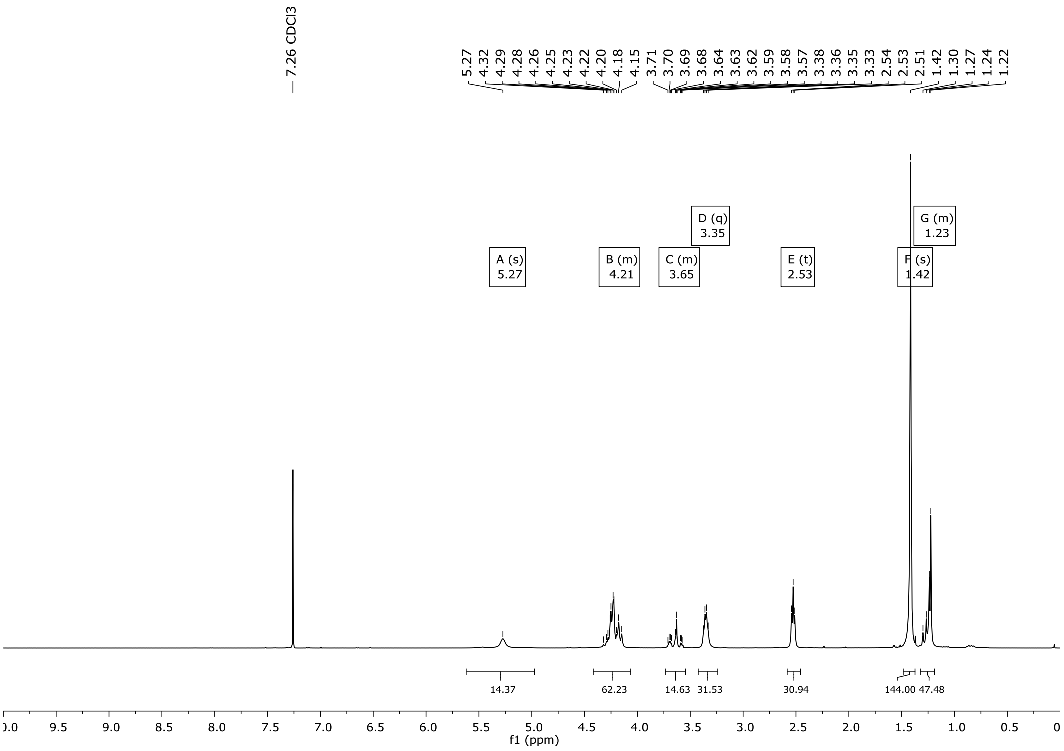

^{13}C NMR (101 MHz, Chloroform-d) OH-TEG-G4-NHBoc

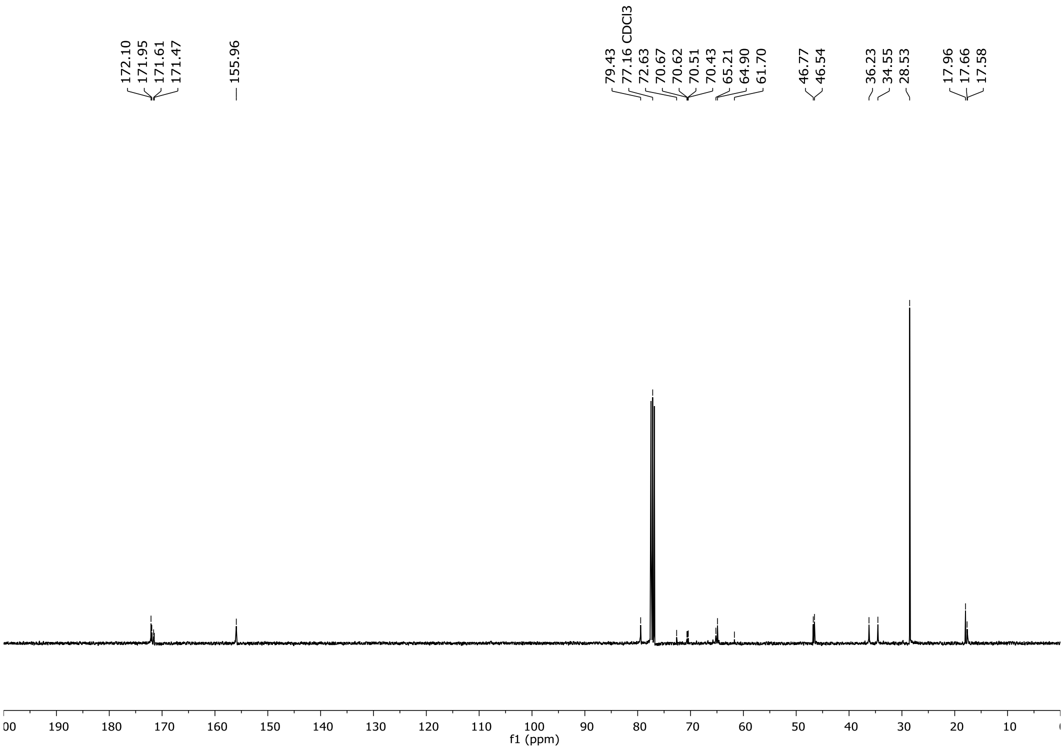

^{1}H NMR (400 MHz, Chloroform-d) RhB-TEG-G4-NHBoc

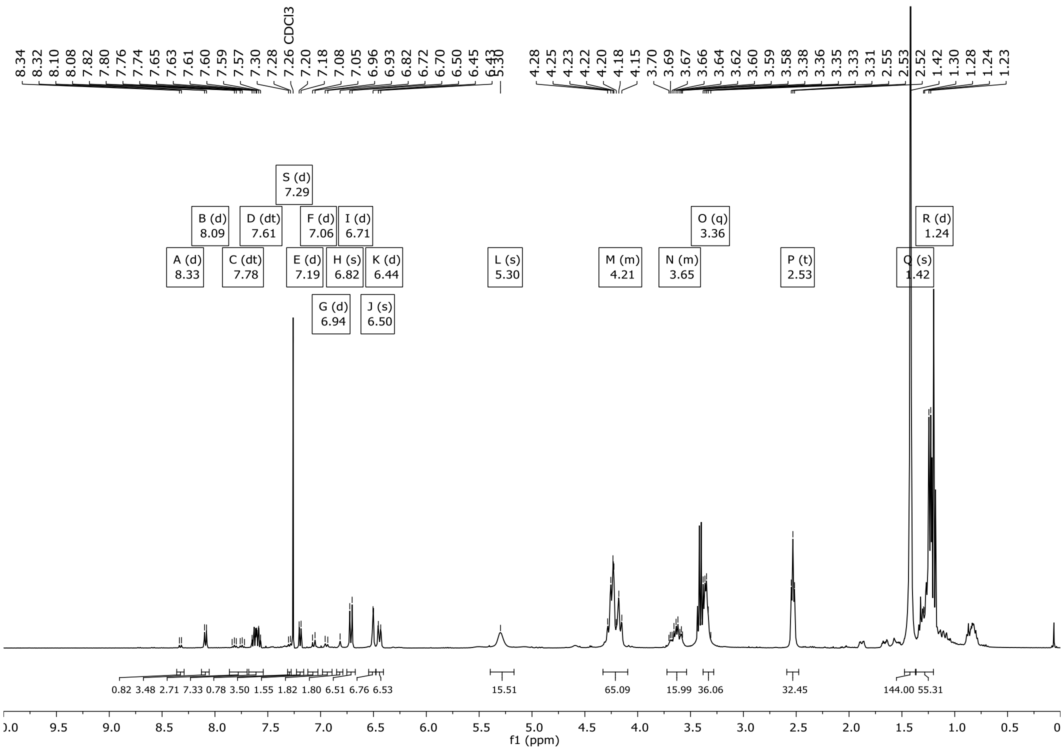

^{13}C NMR (101 MHz, Chloroform-d) RhB-TEG-G4-NHBoc

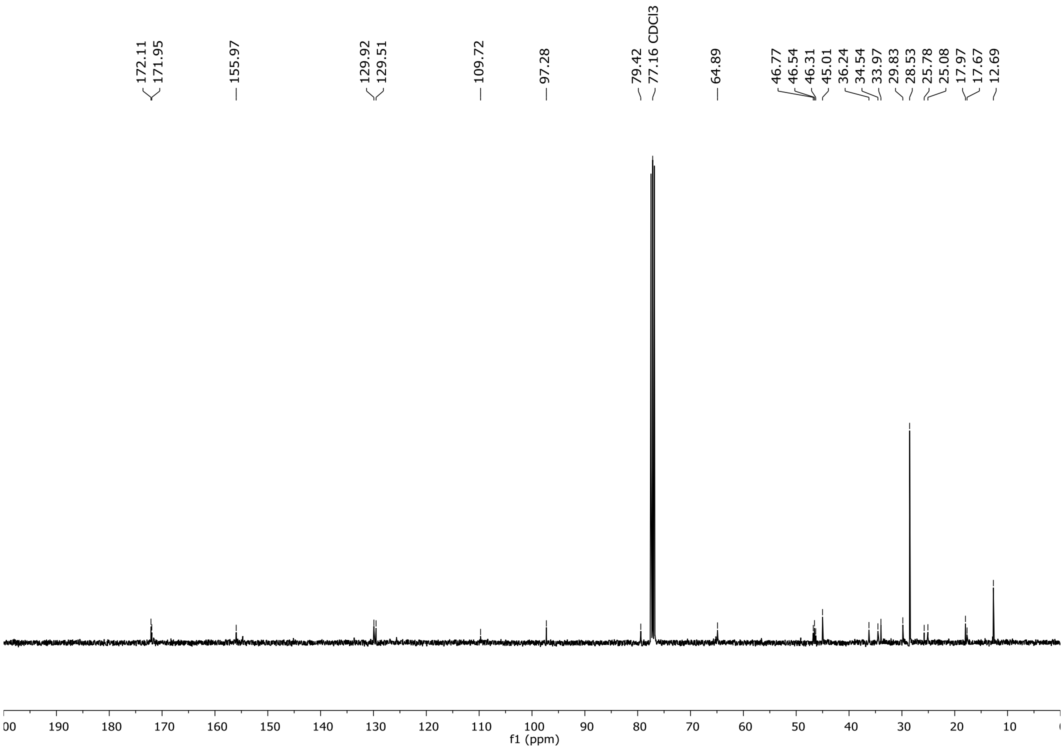

^{1}H NMR (400 MHz, Methanol-d$_4$) RhB-TEG-G4-NH$_3^+$TFA$^-$

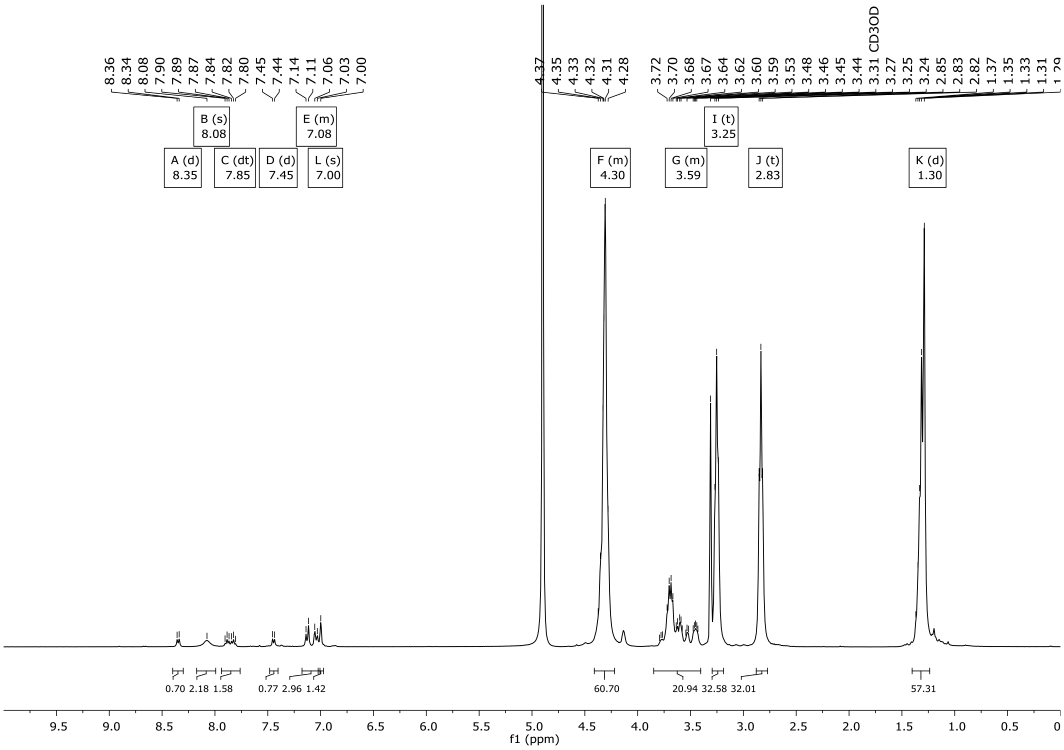

^{13}C NMR (101 MHz, Methanol-d$_4$) RhB-TEG-G4-$NH_3^+TFA^-$

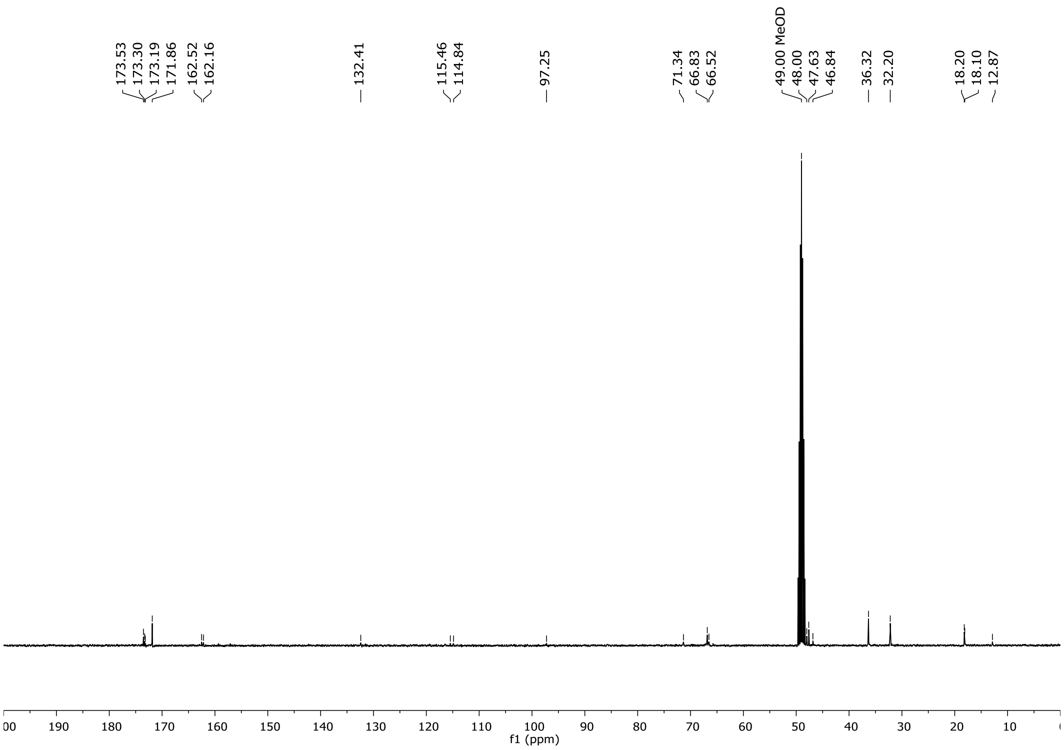

MALDI-spectra

BnTEG-G4-OH

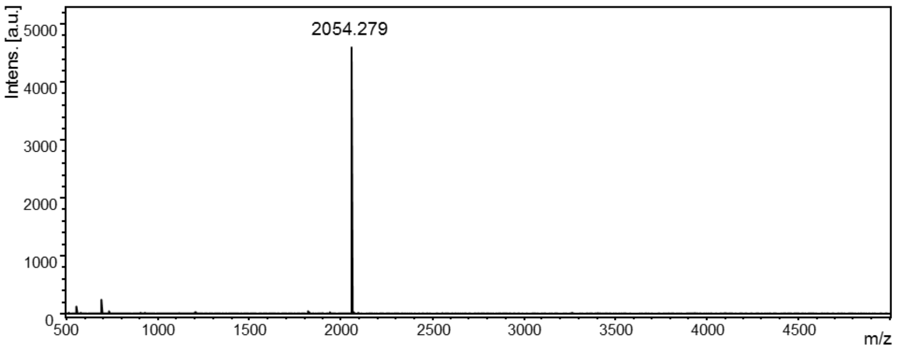

BnTEG-G4-NHBoc

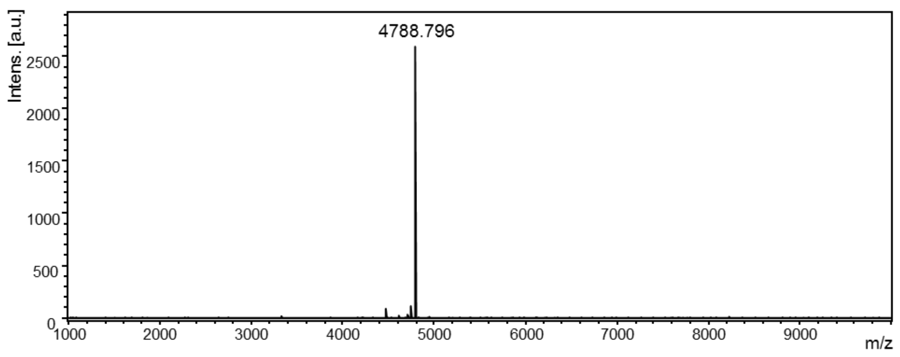

OH-TEG-G4-NHBoc

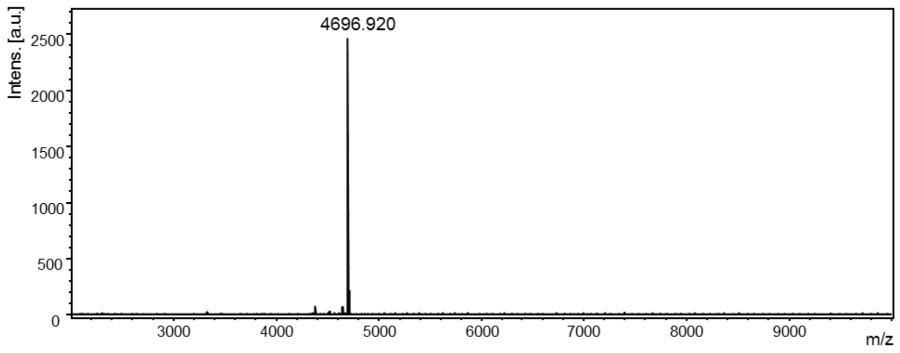

RhB-TEG-G4-NHBoc

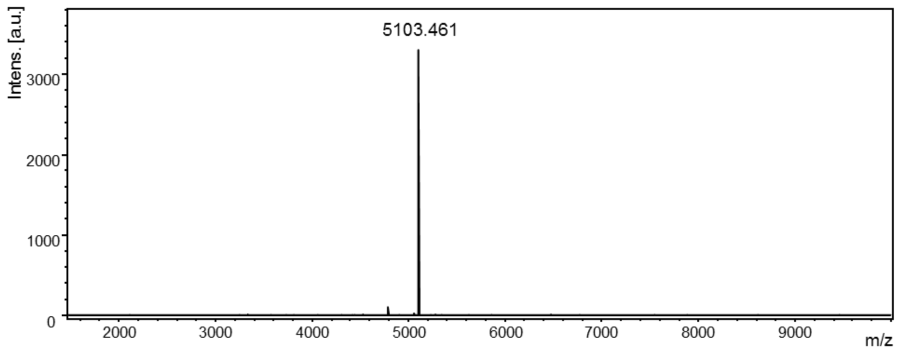

RhB-TEG-G4-$NH_3^+TFA^-$

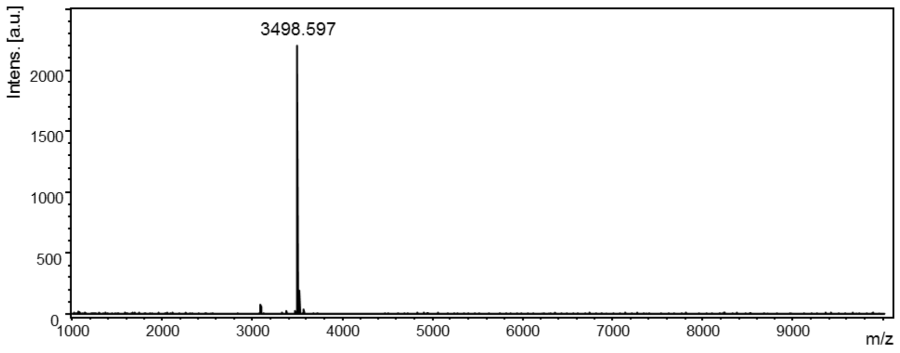

© 2018 by the authors. Submitted for possible open access publication under the terms and conditions of the Creative Commons Attribution (CC BY) license (http://creativecommons.org/licenses/by/4.0/).
